# The FGFR1 Signaling Pathway Upregulates the Oncogenic Transcription Factor FOXQ1 to Promote Breast Cancer Cell Growth

**DOI:** 10.7150/ijbs.74574

**Published:** 2023-01-01

**Authors:** Yan Lin, Fengkang Lin, Zhuoran Zhang, Lijia Peng, Wenli Yang, Mao Yang, Bo Luo, Ting Wu, Dabing Li, Xuesen Li, Bing Ran, Songyot Anuchapreeda, Rujirek Chaiwongsa, Pinyaphat Khamphikham, Suwit Duangmano, Jianming Xu, Tao He, Sakorn Pornprasert

**Affiliations:** 1 Institute for Cancer Medicine and School of Basic Medical Sciences, Southwest Medical University, Luzhou, Sichuan, 646000, China.; 2 Department of Medical Technology, Faculty of Associated Medical Sciences, Chiang Mai University, Chiang Mai, 50200, Thailand.; 3 Department of Physiology, School of Basic Medical Sciences, Southwest Medical University, Luzhou Sichuan, 646000, China.; 4 Functional laboratory, School of Basic Medical Sciences, Southwest Medical University, Luzhou Sichuan, 646000, China.; 5 Department of Molecular and Cellular Biology, Baylor College of Medicine, Houston, Texas 77030, USA.

**Keywords:** gene regulation, breast cancer, FGFR1, ERK2, c-FOS, FOXQ1

## Abstract

FGFR1 is a receptor tyrosine kinase deregulated in certain breast cancers (BCs) with a poor prognosis. Although FGFR1-activated phosphorylation cascades have been mapped, the key genes regulated by FGFR1 in BC are largely unclear. FOXQ1 is an oncogenic transcription factor. Although we found that activation of FGFR1 robustly upregulated FOXQ1 mRNA, how FGFR1 regulates *FOXQ1* gene expression and whether FOXQ1 is essential for FGFR1-stimulated cell proliferation are unknown. Herein, we confirmed that activation of FGFR1 robustly upregulated FOXQ1 mRNA and protein in BC cells. Knockdown of FOXQ1 blocked the FGFR1 signaling-stimulated BC cell proliferation, colony formation, and xenograft tumor growth. Inhibition of MEK or ERK1/2 activities, or knockout of ERK2 but not ERK1 suppressed the FGFR1 signaling-promoted *FOXQ1* gene expression. Inhibition of ERK2 in ERK1 knockout cells blocked, while ectopic expression of FOXQ1 in ERK2 knockout cells rescued the FGFR1-signaling-promoted cell growth. Mechanistically, c-FOS, an early response transcription factor upregulated by the FGFR1-MEK-ERK2 pathway, bound to the *FOXQ1* promoter to mediate the FGFR1 signaling-promoted FOXQ1 expression. These results indicate that the FGFR1-ERK2-c-FOS-FOXQ1 regulatory axis plays an essential role in the FGFR1 signaling-promoted BC growth. Targeting ERK2 and FOXQ1 should block BC growth caused by a deregulated FGFR1 signaling.

## Introduction

Breast cancer has become the most common cancer in the world since 2020 **1.** Although the survival rate of breast cancer patients keeps improving owing to advances in cancer biology, early diagnosis, and new therapies, many patients still die of breast cancer because of our insufficient knowledge about breast cancer growth and unsatisfactory clinical treatment for controlling the growth of heterogeneous breast cancer cells**.** Breast cancer cell growth is supported by many deregulated signaling pathways, including growth factors and their receptor tyrosine kinase**-**signaling pathways**.** The fibroblast growth factor receptor 1 **(**FGFR1**)** is amplified, mutated, or rearranged in 18**%** of breast cancers **2.** Enhanced FGFR1 signaling stimulates cell proliferation, increases cell plasticity and invasiveness, and makes cancer cells resistant to chemotherapy **3-5.** Human breast tumors with FGFR1 overexpression also exhibited a poor prognosis **6.** Previous studies have demonstrated that blockade of the FGF**/**FGFR signaling pathway makes certain cancer cells that are resistant to paclitaxel or inhibitors of EGFR, MET, or VEGFR regain sensitivities to these drugs **7-10.** Multiple clinical trials that use FGFR inhibitors for breast cancer treatment are currently undergoing **11.** However, no FGFR1 inhibitors have been approved for breast cancer treatment, possibly due to their side effects and cancer cell**-**acquired resistance to these inhibitors **11.** Characterizing the specific downstream signaling pathways of FGFR1 and these pathway**-**regulated key genes responsible for mediating FGFR1**-**promoted breast cancer growth will help to identify alternative molecular targets for developing new therapies**.**

FGFR1 is a transmembrane tyrosine kinase receptor of fibroblast growth factors **(**FGFs**).** Upon FGF binding, FGFR1 undergoes dimerization and autophosphorylation to initiate phosphorylation cascades, leading to the activation of multiple downstream signaling pathways, such as the Ras**/**Raf**-**MEK**-**MAPKs, PI3K**-**AKT, PLC**γ-**PKC, and STAT pathways **12-14.** These downstream pathways allow FGFR1 to exert its pleiotropic functions in regulating cell proliferation, differentiation, metabolic homeostasis, and pathogenesis **12-14.** In addition to the direct regulation of cellular function by protein phosphorylation, these downstream FGFR1 signaling pathways regulate cellular functions by regulating gene expression**.** For example, activation of FGFR1 signaling can upregulate TNFAIP3 to promote the proliferation and tumorigenesis of premalignant human mammary epithelial cells **15.** Moreover, FGFR1 increases Gli2 and SOX2 expressions to promote stemness, proliferation, epithelial**-**to**-**mesenchymal transition **(**EMT**)**, and metastasis of non**-**small cell lung cancer **(**NSCLC**)** cells **16, 17.** FGFR1 overexpression and activation also upregulate ZEB**-**1 to promote EMT and EGFR inhibitor resistance in NSCLC cells **18**, and increase the release of inflammatory chemokines such as CXCL1**/**5 to promote mammary tumor growth and progression **19.** Although it is known that FGFR1 signaling can regulate many genes, the underlying molecular mechanisms and the key genes that mediate FGFR1**-**promoted breast cancer cell proliferation are still unclear**.**

The forkhead Q1 **(**FOXQ1**)** is a transcription factor in the forkhead box gene superfamily**.** FOXQ1 is implicated in many biological processes, such as embryonic stem cell development, metabolism, cell senescence, and hair follicle differentiation **20.** Overexpressed FOXQ1 promotes cell proliferation and the development of liver cancer **21**, neuroblastoma **22**, and colorectal cancer **23, 24.** Ectopic expression of FOXQ1 in breast cancer cells and NSCLC cells promotes EMT by repressing E**-**cadherin expression and enhancing vimentin expression **25, 26.** FOXQ1 also upregulates ZEB2 and versican V1 to enhance the invasiveness of hepatocellular carcinoma cells **27** and the stemness and drug resistance of breast cancer cells **28.** Given these oncogenic roles, FOXQ1 has been considered a protooncogene **29-31.** Our previous study demonstrated that the FOXQ1 mRNA is one of 946 mRNAs that are significantly upregulated upon the activation of FGFR1 signaling **15.** However, it remains unclear whether the FGFR1 signaling pathway can promote breast cancer growth by regulating FOXQ1 expression**.**

In this study, we discovered that activation of FGFR1 signaling significantly upregulates the oncogenic transcription factor FOXQ1 through the FGFR1**-**ERK2**-**c**-**FOS gene regulatory axis in breast cancer cells**.** Knockdown of FOXQ1 expression effectively prevented FGFR1**-**promoted breast cancer cell growth in culture and tumor growth *in vivo***.**

## Materials and Methods

**Cell culture.** MCF10DCIS**.**com **(**DCIS**.**COM**)** cell line was described previously **32.** This cell line was derived from a cell culture of a lesion formed by xenotransplantation of the premalignant MCF10AT human breast epithelial cells with Ras expression**.** DCIS**-**iFGFR1 cell line carrying an integrated iFGFR1 expression vector in their genomes and the matched DCIS**-**Ctrl control cell line carrying an integrated empty vector were generated from DCIS**.**COM cells as described previously **15.** The iFGFR1 construct was initially invented by Freeman et al**.**, 2003 and used as a molecular tool for specific activation of the FGFR1 signaling **33.** The iFGFR1 expressed in DCIS**-**iFGFR1 cells consists of the v**-**Src myristoylation membrane**-**targeting sequence, FGFR1 cytoplasmic domain, and the FKBP12 dimerization domain **15, 33.** AP20187 binding to the FKBP12 dimerization domain induces iFGFR1 dimerization and activation, leading to the activation of FGFR1 downstream signaling pathways and an increase in DCIS**-**iFGFR1 cell proliferation **15.** DCIS**-**iFGFR1 cell lines with CRISPR**/**Cas9 mediated ERK1 or ERK2 knockout cell lines were also described previously **15.** These DCIS**.**COM cell**-**derived cell lines were cultured in DMEM**/**F12 **(**1**:**1**)** medium containing 5**%** horse serum**.** The MDA**-**MB**-**231 human triple**-**negative breast cancer cell line was purchased from the Shanghai Cell Bank of the Chinese Academy of Sciences, and cultured in a high**-**glucose DMEM medium with 10**%** fetal bovine serum **(**FBS**).** HEK293 cells were cultured in a high**-**glucose DMEM medium containing 10**%** FBS**.** All cells were cultured at 37°C in a tissue culture incubator with 5**%** CO2**.**

**Western blot analysis.** Total protein samples were extracted from cells using a cell lysis buffer containing 50 mM Tris **(**pH 7**.**4**)**, 150 mM NaCl, 1**%** Triton X**-**100, 1**%** sodium deoxycholate, 0**.**1**%** SDS, and mixed phosphatase and proteinase inhibitors including sodium orthovanadate, sodium fluoride, EDTA, and leupeptin **(**P0013B, Beyotime**).** Cytoplasmic proteins and nucleoproteins were prepared using the Nuclear and Cytoplasmic Protein Extraction Kit **(**P0028, Beyotime**).** Total protein **(**50 μg**)**, cytoplasmic protein **(**50 μg**)**, or nuclear protein **(**20 μg**)** samples were separated in 4-16**%** precast SDS**-**PAGE gels **(**RTG1010G16, Rosetta Stone**)** and transferred onto a polyvinylidene fluoride membrane **(**PVDF**) (**ISEQ00010, Millipore**).** Membranes were blocked with 5**%** w**/**v fat**-**free dry milk dissolved in Tris**-**buffered saline **(**pH 8**.**3**)** plus 0**.**1**%** Tween**-**20 **(**TBS**-**T**)** at room temperature for 1 hour**.** A primary antibody against FGFR1 **(**9740s, cell Signaling Technology**)**, p**-**FGFR1 **(**3471, Cell Signaling Technology**)**, FOXQ1 **(**sc**-**166264, Santa Cruz**)**, c**-**FOS **(**sc**-**271243, Santa Cruz; 2250s, Cell Signaling Technology**)**, ERK1**/**2 **(**9102S, Cell Signaling**)**, phospho**-**Thr202**/**Tyr204**-**ERK1**/**2 **(**p**-**ERK1**/**2**) (**9101S, Cell Signaling**)**, β**-**actin **(**3700S, Cell Signaling**)**, tubulin **(**2148S, Cell Signaling**)**, PARP1 **(**13371**-**1**-**AP, Proteintech**)**, or GAPDH **(**60004**-**1**-**Ig, Proteintech**)** was incubated with the membrane at 4°C overnight**.** After washing with TBS**-**T, membranes were further incubated with a fluorescence**-**labeled secondary antibody, including DyLight 800**-**conjugated anti**-**rabbit IgG **(**5151P, Cell Signaling**)** or DyLight 800**-**conjugated anti**-**mouse IgG **(**5257P, Cell Signaling**)**, for 2 hours at room temperature**.** The fluorescence intensity of bands was imaged using the Odyssey Imaging System **(**LI**-**COR**).** Band intensity was quantitatively analyzed using the software ImageJ**.** Band intensity of β**-**actin, tubulin, or GAPDH served as an internal loading control of total or cytoplasmic proteins**.** The band intensity of PARP1 served as an internal loading control of nuclear proteins**.**

**Reverse transcription and quantitative PCR (RT-qPCR).** Total RNA samples were isolated from cells and reversely transcribed into cDNA, as described previously **15.** TaqMan fluorescence qPCR was performed as described previously **15.** Probe #6 in the Universal Probe Library **(**04688970001, Roche**)**, the 5**'** forward primer, gcggactttgcactttgaa, and the 3**'** reverse primer, tttaaggcacgtttgatgga, were used for qPCR to measure FOXQ1 mRNA**.** Probe #67 in the same Roche library, the 5**'** forward primer, actaccactcacccgcagac, and the 3**'** reverse primer, ccaggtccgtgcagaagt, were used for qPCR to measure c**-**FOS mRNA**.** The relative expression levels of FOXQ1 and c**-**FOS mRNAs were calculated using the 2^^^**^-^**^ΔΔCt^ method, with parallel measurement of β**-**actin mRNA as an internal control for normalization**.**

**shRNA-mediated stable knockdown of *FOXQ1* expression.** Three psi**-**LVRU6GP plasmids with an eGFP expression cassette were purchased from GeneCopoeia **(**Rockville, MD**).** One plasmid for expressing the shRNA coding sequence of gcggactttgcactttgaatc **(**shFOXQ1#1**)** and another plasmid for expressing the shRNA coding sequence of ggagtatttaaacttagtcca **(**shFOXQ1#2**)** were used to make FOXQ1 knockdown DCIS**-**iFGFR1 and MDA**-**MB**-**231 cell pools**.** The other plasmid with a non**-**targeting shRNA coding sequence was used as a control**.** Cells were transfected with each plasmid DNA using Lipofectamine 8000 **(**c0533, Beyotime Biotechnology**)**, followed by culturing the cells in a growth selection medium containing 2**.**25 μg**/**ml puromycin for two weeks**.** The eGFP**-**positive cells were selected from the survived cells by flow cytometry and expanded**.** The knockdown efficiency of FOXQ1 mRNA and protein were examined by RT**-**qPCR and Western blot analysis**.**

**Cell growth and colony formation assays.** For cell growth assay, cells were seeded in 96**-**well plates with 2000 cells / well, cultured overnight, and treated in media containing 0**.**02**%** DMSO **(**vehicle**)** or 100 nM AP20187 for 24, 48, or 72 hours**.** Cell viability was assayed using a Cell Counting Kit**-**8 **(**CCK**-**8**) (**K1018, Dojindo**)** on days 1, 2, and 3**.** Briefly, cells were incubated with CCK**-**8 reagent containing 10**%** of 2**-(**2**-**methoxy**-**4**-**nitrophenyl**)-**3**-(**4**-**nitrophenyl**)-**5**-(**2,4**-**disulfonic acid benzene**)-**2H**-**tetrazolium monosodium salt **(**WST**-**8**)** at 37°C in a humidified incubator for 2 hours**.** The optical density **(**OD**)** values were measured at 450 nm**.** The increase of relative cell number was presented as the 450 nm light absorbance ratio at the experimental ending**-**time point to that of the experimental starting**-**time point**.** For the colony formation assay, 600 cells were plated in 6**-**well plates and cultured for 11 days until visible clones formed**.** The cells were fixed in 4**%** methanol for 15 minutes and stained with crystal violet for 30 minutes**.** Cells were washed several times with water**.** Cellular colonies were imaged, and the colonies consisting of more than 50 cells were counted**.**

**Xenograft tumor growth assay.** Four**-**week**-**old female BALB**/**c**-**nu mice were purchased from Beijing Huafukang Biosciences in China**.** For each injection site, two million control DCIS**-**iFGFR1 cells with the non**-**targeting sh**-**NC expression or FOXQ1**-**knockdown DCIS**-**iFGFR1 cells with the sh**-**FOXQ1**-**1 or sh**-**FOXQ1**-**2 shRNA expression in 0**.**1 ml of PBS, pH 7**.**45 were orthotopically injected into a mammary gland fat pad of 6**-**week**-**old female BALB**/**c**-**nu mouse on day 1**.** Eight mice were used in each group**.** From day 3 to 25, the mice with the injected cells were treated with either 50 μl of saline containing 0**.**4**%** ethanol **(**vehicle**)** or AP20187 **(**1 mg**/**kg**)** per mouse **(**i**.**p**.**, once every other day**).** On day 25, all mice were euthanized, and the xenograft tumors in these mice were dissected and collected**.** The individual tumors were imaged and weighed**.** The animal protocol was approved by The Institutional Animal Care and Use Committee of Southwest Medical University**.**

**Immunohistochemistry (IHC).** Fresh tumor samples from mice were fixed in 4**%** paraformaldehyde in PBS, pH 7**.**45 dehydrated in a series of solutions with ascending ethanol concentrations, and embedded in paraffin**.** Tissue sections prepared from paraffin**-**embedded tissues were dewaxed in xylene, and rehydrated in a series of solutions with descending ethanol concentrations and ddH_2_O**.** Antigens were retrieved by heating the sections at 170°C for 4 minutes in a citrate buffer **(**10 mM**).** The endogenous peroxidase activity was inactivated by incubating the sections in a 3**%** hydrogen peroxide solution for 15 minutes**.** The sections were incubated with a goat serum**-**containing blocking solution **(**Cat # abs933, Absinand**),** and then with the rabbit anti**-**FOXQ1 antibody **(**1**:**125, SAB2107907, Sigma**-**Aldrich**)** or the rabbit anti**-**ki67 antibody **(**1**:**400, 9027, Cell signaling**)** overnight at 4°C**.** The sections were then incubated with one drop of SignalStain^®^ Boost Detection Reagent **(**Rabbit, 8114, Cell Signaling**)** containing horseradish peroxidase **(**HRP**)-**conjugated anti**-**rabbit IgG antibody at room temperature for 2 hours**.** The bound HRP activity was visualized by incubating the sections with the DAB substrate for 3 minutes**.** Finally, the sections were counterstained with hematoxylin for 30 seconds**.** FOXQ1 immunoreactivity was scored according to the percentage of positive cell numbers **(**< 25**%**, 1 score; 25**-**49**%**, 2 scores; > 50**%**, 3 scores**)**, and the staining intensity **(**negative, 0 score; weak, 1 score; moderate, 2 scores; strong, 3 scores**).** The total score **=** positive cell **%** score × staining intensity score**.** The percentage of Ki67**-**positive tumor cells to total tumor cells was determined by measuring the Ki67**-**positive tumor cell area and the total tumor cell area, then calculating the ratio of the Ki67**-**positive tumor cell area to the total tumor cell area using the Image J software**.**

**siRNA-mediated knockdown and transient expression of FOXQ1.** To knock down FOXQ1, ERK1 knockout DCIS**-**iFGFR1 cells were seeded in a 96**-**well plate with 3000 cells / well and cultured overnight**.** After replacing the culture medium with a serum**-**free medium, cells were transfected with 4 pmol**/**well of the siFOXQ1**-**1 siRNA, the siFOXQ1**-**2 siRNA, or a scrambled non**-**targeting siRNA **23 (**Supplemental Table S1**)** using Lipofectamine 8000**.** To express FOXQ1, ERK2 knockout DCIS**-**iFGFR1 cells were transfected with 200 ng**/**well DNA of the FOXQ1 expression vector pEZ**-**LV201 **(**EX**-**Y5225**-**LV201, GeneCopoeia^TM^**)** or the matched empty control vector LV201CT **(**EX**-**NEG**-**LV201, GeneCopoeia^TM^**)** using Lipofectamine 8000**.** Six hours later, the medium with the transfection reagents was replaced with a fresh growth medium**.** Cells in different groups were further cultured for 6, 30, 54, or 78 hours before the cell viability was assayed using the CCK**-**8 kit**.**

**Bioinformatics analysis.** The 5**'** regulatory sequence of the human FOXQ1 gene was downloaded from the genome browser in the UCSC database**.** The DNA sequence from the **-**2000^th^ bp to the 100^th^ bp from the transcriptional start site **(**TSS**)** that contains the FOXQ1 promoter was input to the PROMO, a virtual laboratory based on Version 8**.**3 of the TRANSFAC database **34, 35**, to predict transcription factor**-**binding motifs**.** The maximum matrix dissimilarity rate was set to 5**%** or less**.** The R language package **"**cluster profile**"** was used to conduct gene ontology **(**GO**)** and Kyoto Encyclopedia of Genes and Genomes **(**KEGG**)** analysis for the predicted transcription factors**.**

**Chromatin immunoprecipitation (ChIP) assay.** DCIS**-**iFGFR1 cells in 10**-**cm culture dishes were treated with 0**.**02**%** DMSO **(**vehicle**)** or 100 nM AP20187 for 1 hour**.** MDA**-**MB**-**231 cells in 10**-**cm culture dishes were treated with vehicle **(**1 μl H2O**/**ml medium**)** or 100 ng**/**ml bFGF and 20 μg**/**ml heparin for 1 hour**.** The treated cells were used for ChIP assays by following the manufacturer's protocol of a ChIP kit **(**ab500, Abcam**).** Briefly, DNA and associated proteins in cells were cross**-**linked with 1**.**1**%** of formaldehyde for 10 minutes at room temperature**.** The cross**-**linking reaction was quenched with 0**.**125 M glycine for 5 minutes**.** The cross**-**linked DNA**-**protein complexes were sheared by sonication into 200**-**1000 bp DNA fragments**.** Sonicated lysates were subjected to ChIP using a c**-**FOS antibody or non**-**immune IgG **(**a negative control**).** The precipitated DNA**-**protein complexes were treated to reverse crosslink**.** qPCR was performed using the SYBRTM Green PCR Master Mixes kit **(**4368577, life technology**)** to measure the DNA amount of the FOXQ1 promoter region containing a predicted c**-**FOS binding motif**.** A forward primer **(**5**'-**gccccaggggaagaggaggacg**)** and a reverse primer **(**5**'-**atgggctccgactttcactttt**)** were used in the qPCR assay**.**

**Luciferase reporter assay.** The DNA fragment of the human *FOXQ1* gene promoter from the **-**1000^th^ 5**'** sequence to the 1^st^ bp of exon 1 with the predicted c**-**FOS binding motif was cloned into the pGL3**-**basic vector to generate the luciferase reporter driven by the wild**-**type *FOXQ1* gene promoter**.** This reporter was designated as FOXQ1**-**WT**-**Luc reporter**.** The other luciferase reporter, FOXQ1**-**MUT, was constructed by deleting the predicted c**-**FOS binding motif from the **-**817^th^ bp to the **-**808^th^ bp in the same 5**'** *FOXQ1* promoter DNA fragment**.** The pRL**-**SV40**-**N plasmid, a Renilla luciferase expression vector **(**D2762, Beyotime Biotechnology**)**, was used in a co**-**transfection dual luciferase assay**.** HEK293 cells with 50**%** confluence in a 24**-**well plate were co**-**transfected with 50 ng of pRL**-**SV40**-**N plasmid DNA and 450 ng of pGL**-**basic, FOXQ1**-**WT, or FOXQ1**-**MUT plasmid DNA using the Lipofectamine 8000**.** After 24 hours, the transfected cells were treated with 0**.**02**%** DMSO **(**vehicle**)**, 200 nM 12**-**O**-**tetradecanoylphorbol**-**13**-**acetate **(**TPA, 4174S, Cell Signaling**)**, or 200 nM TPA plus 10 μM T**-**5244, a c**-**FOS inhibitor **(**HY**-**75954, MedChemExpress**).** In another experiment, the transfected cells were treated with vehicle **(**the culture medium**)** or 100 ng**/**ml bFGF plus 20 μg**/**ml heparin for 1 and 3 hours**.** Luciferase activities were measured using the dual luciferase reporter system **(**RG042, Beyotime Biotechnology**).** Renilla luciferase activity was used as an endogenous control for normalizing the transfection efficiencies among different groups**.**

**Statistical analysis.** The statistical analyses were carried out with GraphPad Prism 8 software**.** Data are presented as the mean ± SD**.** The differences between the two groups were analyzed using Student**'**s t**-**test**.** The differences among three or more groups were analyzed using One**-**Way ANOVA**.** A P**-**value less than 0**.**05 was considered statistically significant in all analyses**.**

## Results

### Activation of FGFR1 signaling upregulates FOXQ1 expression

**Figure 1:**
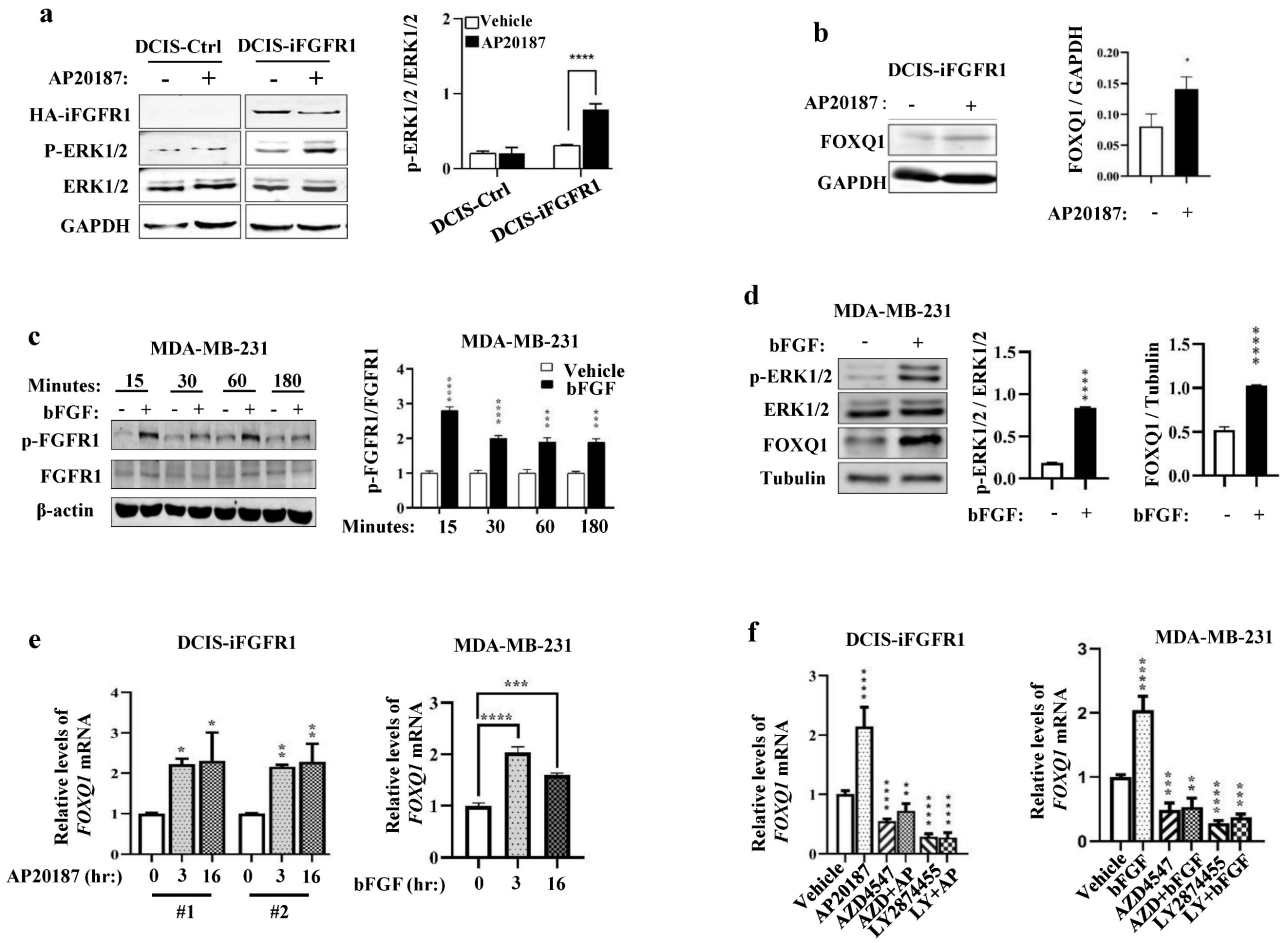
** Activation of FGFR1 signaling upregulates FOXQ1 expression. a.** Western blot (WB) analysis. AP20187 treatment (100 nM) increased the levels of p-ERK1/2 in DCIS-iFGFR1 cells, but not in DCIS-Ctrl cells. GAPDH served as a loading control. The average ratio of p-ERK1/2 to total ERK1/2 band intensities obtained from 3 assays are presented in the bar graph. **b.** WB analysis. AP20187 treatment increased FOXQ1 in DCIS-iFGFR1 cells. The average ratio of FOXQ1 to GAPDH band intensities obtained from 3 assays are presented in the bar graph. **c.** WB analysis. bFGF treatment (100 ng/ml) increased p-FGFR1 in MDA-MB-231 cells at the time points indicated. β-actin served as a loading control. The average ratio of p-FGFR1 to total FGFR1 band intensities obtained from 3 assays are presented in the bar graph. **d.** WB analysis. bFGF treatment increased p-ERK1/2 and FOXQ1 in MDA-MB-231 cells. Tubulin served as a loading control. The average ratios of p-ERK1/2 to total ERK1/2, and FOXQ1 to tubulin band intensities are presented in the bar graphs. **e.** DCIS-iFGFR1-1/2 (#1 and #2) and MDA-MB-231 cells were treated with AP20187 and bFGF, respectively, for the time points indicated. The FOXQ1 mRNA was measured by RT-qPCR in 3 independent samples, and normalized to β-actin mRNA. **f.** DCIS-iFGFR1 and MDA-MB-231 cells were treated with vehicle, AP20187, bFGF, AZD4547 (AZD, 500 nM), and/or LY2874455 (LY, 500 nM) as indicated. The relative level of FOXQ1 mRNA was measured as described in Panel e. Data in all bar graphs are presented as Mean ± SD. ^*^, ^**^, ^***^, ^****^, *p* < 0.05, 0.01, 0.001, 0.0001, respectively versus the vehicle-treated control groups, which were analyzed by One-Way ANOVA.

In DCIS**-**iFGFR1 cells with iFGFR1 expression, RNA**-**Seq analysis revealed that activation of FGFR1 signaling by AP20187**-**induced dimerization changed the expression levels of many genes **15.** The *FOXQ1* gene is one of the significantly upregulated genes upon activation of the FGFR1 signaling in DCIS**-**iFGFR1 cells **15.** Because the *FOXQ1* gene is a protooncogene **25, 36, 37** that has not been studied in FGFR1**-**regulated cell growth, we aimed to investigate how FGFR1 signaling upregulates FOXQ1 expression and whether FOXQ1 is responsible for mediating breast cancer cell growth promoted by FGFR1 signaling**.** As predicted, the levels of phosphorylated ERK1**/**2 **(**p**-**ERK1**/**2**)**, the downstream MAPKs of the FGFR1 signaling pathway, were increased in AP20187**-**treated DCIS**-**iFGFR1 cells **(**line #1**)** with HA**-**iFGFR1 protein expression but not in DCIS**-**Ctrl cells without HA**-**iFGFR1 protein expression, indicating that AP20187 treatment activated FGFR1 signaling in DCIS**-**iFGFR1 cells **(**Fig**.** 1a**).** The AP20187**-**activated FGFR1 signaling in DCIS**-**iFGFR1 cells significantly upregulated FOXQ1 protein expression **(**Fig**.** 1b**).** In MDA**-**MB**-**231 breast cancer cells with endogenous FGFR1 expression **38**, bFGF treatment activated FGFR1 as reflected by its phosphorylation on Tyr653**/**654 measured at different time points **(**Fig**.** 1c**)**, which was associated with robustly increased p**-**ERK1**/**2 and FOXQ1 protein expression **(**Fig**.** 1d**).** Furthermore, the FGFR1 signaling activated by AP20187 and bFGF treatments also significantly increased FOXQ1 mRNA expression in DCIS**-**iFGFR1 cells **(**lines #1 and #2**)** and MDA**-**MB**-**231 cells, respectively **(**Fig**.** 1e**).** Furthermore, inhibition of FGFR function by FGFR inhibitors, AZD4547 **39** and/or LY2874455 **40**, not only abolished AP20187**-** or bFGF**-**induced FOXQ1 mRNA expression but also further downregulated the basal expression level of FOXQ1 mRNA as compared to the FOXQ1 mRNA expression level in vehicle**-**treated DCIS**-**iFGFR1 **(**line #1**)** and MDA**-**MB**-**231 cells **(**Fig**.** 1f**).** Together, these results demonstrate that activation of FGFR1 signaling significantly upregulates FOXQ1 mRNA and protein expression**.**

### FOXQ1 upregulation is required for FGFR1 signaling-promoted cell growth and colony formation

**Figure 2:**
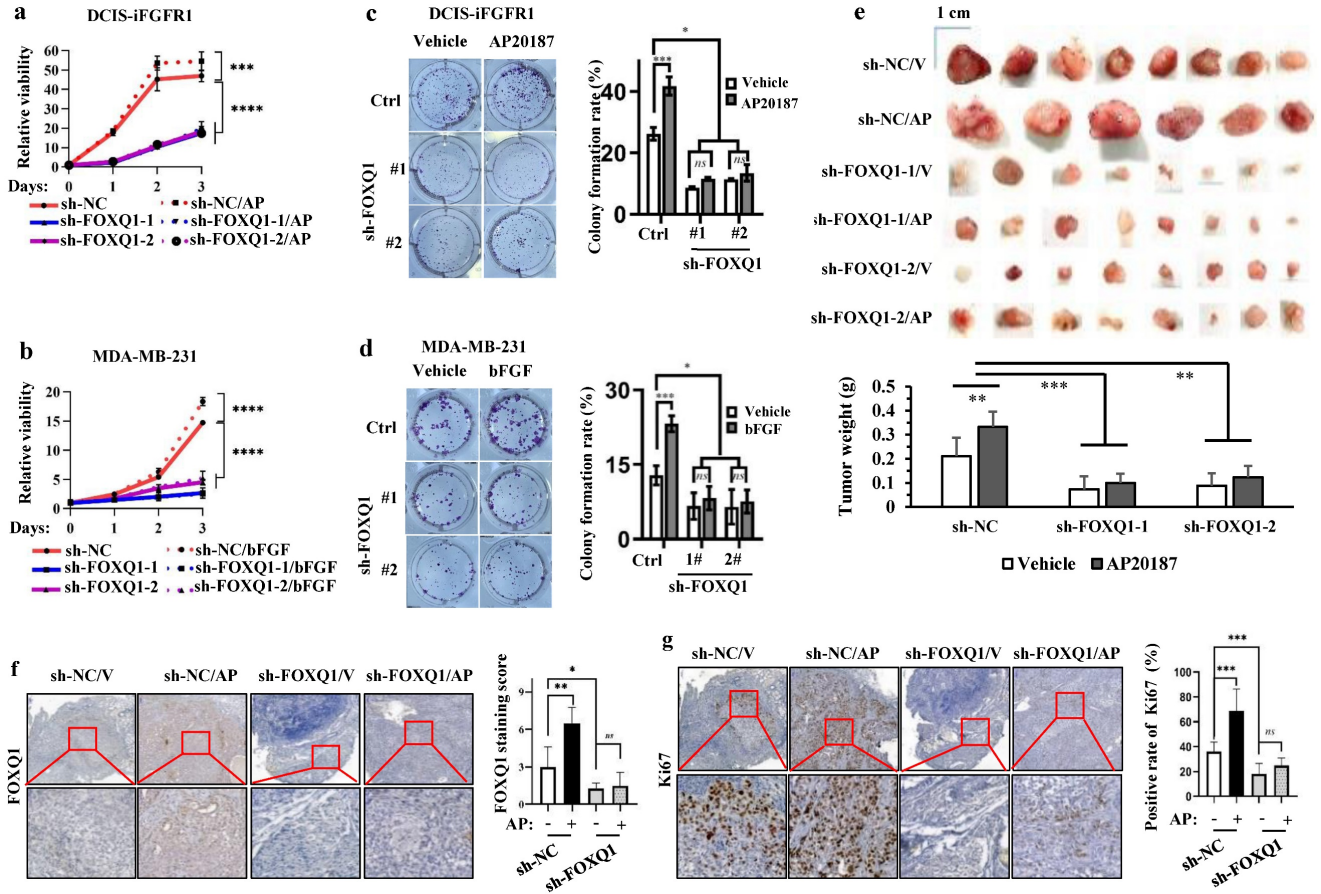
** FOXQ1 plays an essential role in the FGFR1-signaling pathway-promoted breast cancer cell growth, colony formation and tumor growth. a and b.** AP20187 and bFGF treatments stimulated the proliferation of DCIS-iFGFR1 and MDA-MB-231 cells with the non-targeting sh-NC expression. Knockdown of FOXQ1 in DCIS-iFGFR1 and MDA-MB-231 cells with sh-FOXQ1-1/2 expression strongly decreased cell proliferation and diminished the growth responses of these cells to AP20187 and bFGF treatments, respectively. The data were obtained from 12 independent biological samples in each group. **c and d.** Colony formation assay. DCIS-iFGFR1 or MDA-MB-231 cells with stable expression of sh-NC control, sh-FOXQ1-1 or sh-FOXQ1-2 shRNA were cultured in 6-well plates (600 cells/well) and treated with vehicle, AP20187 or bFGF as indicated. Cells were stained with crystal violet, and the formed cellular colonies containing more than 50 cells in each well were counted and presented as mean ± SD. **e.** Tumors developed in 25 days from 2 million-injected control DCIS-iFGFR1 cells with sh-NC expression or FOXQ1 knockdown DCIS-iFGFR1 cells with sh-FOXQ1-1 or sh-FOXQ1-2 expression in BALB/c-nu mice (n=8) treated with vehicle (V) or AP20187 (AP) as indicated. The average weight of tumors in each group was presented as Mean ± SD in the bar graph. **f and g.** Representative images of FOXQ1 and Ki67 immunohistochemical staining on the tissue sections prepared from the tumors derived from DCIS-iFGFR1 cells with sh-NC (control), or sh-FOXQ1-1 (knockdown) expression in mice treated with vehicle (V) or AP20187 (AP) as indicated. FOXQ1 immunoreactivity was scored from FOXQ1-stained sections prepared from 6-8 tumors in each group, and average scores are presented. The relative percentages of Ki67-positive tumor cells to total cells were determined from Ki67-stained sections prepared from 6-8 tumors in each group. In all panels, quantitative data were presented as Mean ± SD. ^*^, ^**^, ^***^, and ^****^ indicate *p* < 0.05, 0.01, 0.001, and 0.0001, respectively obtained from the One-Way ANOVA test.

To assess the function of FOXQ1 upregulation in FGFR1 signaling**-**promoted cell growth, we knocked down FOXQ1 expression in DCIS**-**iFGFR1 cells and MDA**-**MB**-**231 cells using two different shRNA**-**expressing vectors**.** FOXQ1 mRNA and protein were markedly reduced in both types of cells with stable expression of either shRNA targeting FOXQ1 mRNA **(**sh**-**FOXQ1**)** compared with the control cell pools with the expression of a non**-**targeting shRNA control **(**sh**-**NC**) (**Supplementary Fig**.** S1a**-**d**).** AP20187 treatment promoted the growth of DCIS**-**iFGFR1 cells with sh**-**NC expression, while the two independent DCIS**-**iFGFR1 cell pools with sh**-**FOXQ1**-**1**/**2**-**mediated knockdown of FOXQ1 expression showed much slower growth rates and failed to respond to AP20187**-**stimulated cell growth **(**Fig**. 2a).** These observations were further validated by comparing the growth rates of sh**-**NC**-**expressing and FOXQ1 knockdown MDA**-**MB**-**231 cells**.** bFGF treatment significantly stimulated the growth of MDA**-**MB**-**231 cells expressing sh**-**NC, while sh**-**FOXQ1**-**1**/**2**-**mediated knockdown of FOXQ1 drastically reduced the growth rates of MDA**-**MB**-**231 cells and completely abolished their growth response to bFGF stimulation **(**Fig**. 2b).** These results indicate that FOXQ1 upregulation is required for FGFR1 signaling**-**promoted cell growth**.**

When cells were seeded at very low densities in culture, about 26**%** of control DCIS**-**iFGFR1 cells and 13**%** of control MDA**-**MB**-**231 cells expressing sh**-**NC grew and formed individual colonies**.** Activation of FGFR1 signaling in DCIS**-**iFGFR1 cells by AP20187 or MDA**-**MB**-**231 cells by bFGF significantly increased the percentages of these cells to form individual colonies**.** However, only about 10**%** of FOXQ1 knockdown DCIS**-**iFGFR1 cells treated with AP20187 and 7**%** of FOXQ1 knockdown MDA**-**MB**-**231 cells treated with bFGF were able to form individual colonies**.** The sizes of these colonies were also much smaller than that formed from control cells **(**Fig**.** 2c and d**).** These results indicate that FOXQ1 upregulated by FGFR1 signaling is required for FGFR1 signaling**-**promoted colony formation of breast cancer cells**.**

### Knockdown of *FOXQ1* expression inhibits FGFR1 signaling-promoted growth of xenograft tumors derived from human breast cancer cells in mice

To examine whether FOXQ1 upregulation is required for FGFR1 signaling**-**promoted breast tumor growth *in vivo*, we injected control DCIS**-**iFGFR1 cells with sh**-**NC expression and FOXQ1**-**knockdown DCIS**-**iFGFR1 cells with sh**-**FOXQ1**-**1**/**2 expression into the mammary gland fat pads of female BALB**/**c**-**nu mice**.** We treated these mice with vehicle or AP20187 via subcutaneous injection and examined tumor sizes and weights on day 25 post cell injection**.** The average size and weight of tumors derived from sh**-**NC**-**expressing DCIS**-**iFGFR1 cells in AP20187**-**treated mice were significantly larger and heavier than that in vehicle**-**treated mice**.** Importantly, the average size and weight of tumors derived from sh**-**FOXQ1**-**1**/**2**-**expressing DCIS**-**iFGFR1 cells in vehicle**-** or AP20187**-**treated mice were several folds smaller and lighter compared with the tumors derived from sh**-**NC**-**expressing DCIS**-**iFGFR1 cells in vehicle**-** or AP20187**-**treated mice **(**Fig**.** 2e**).** Immunostaining assay validated the expected levels of FOXQ1 protein, which was moderate in vehicle**-**treated control tumors, highly induced in AP20187**-**treated control tumors, and very low in both vehicle and AP20187 treated FOXQ1 knockdown tumors **(**Fig**.** 2f**).** In agreement with tumor growth rates, the percentage of Ki67**-**positive proliferating cells in AP20187**-**treated control tumors was significantly higher than that in vehicle**-**treated control tumors, while the percentages of Ki67**-**positive cells in both vehicle**-** and AP20187**-**treated FOXQ1**-**knockdown tumors were significantly lower than that in both vehicle**-** and AP20187**-**treated control tumors **(**Fig**.** 2g**).** These results demonstrate that FOXQ1 plays an important role in the growth of DCIS**-**iFGFR1 cell**-**derived tumors; and FOXQ1 upregulation is required for FGFR1 signaling**-**promoted breast tumor cell proliferating and tumor growth *in vivo***.**

### ERK2 is required for FOXQ1 upregulation stimulated by FGFR1 signaling

**Figure 3:**
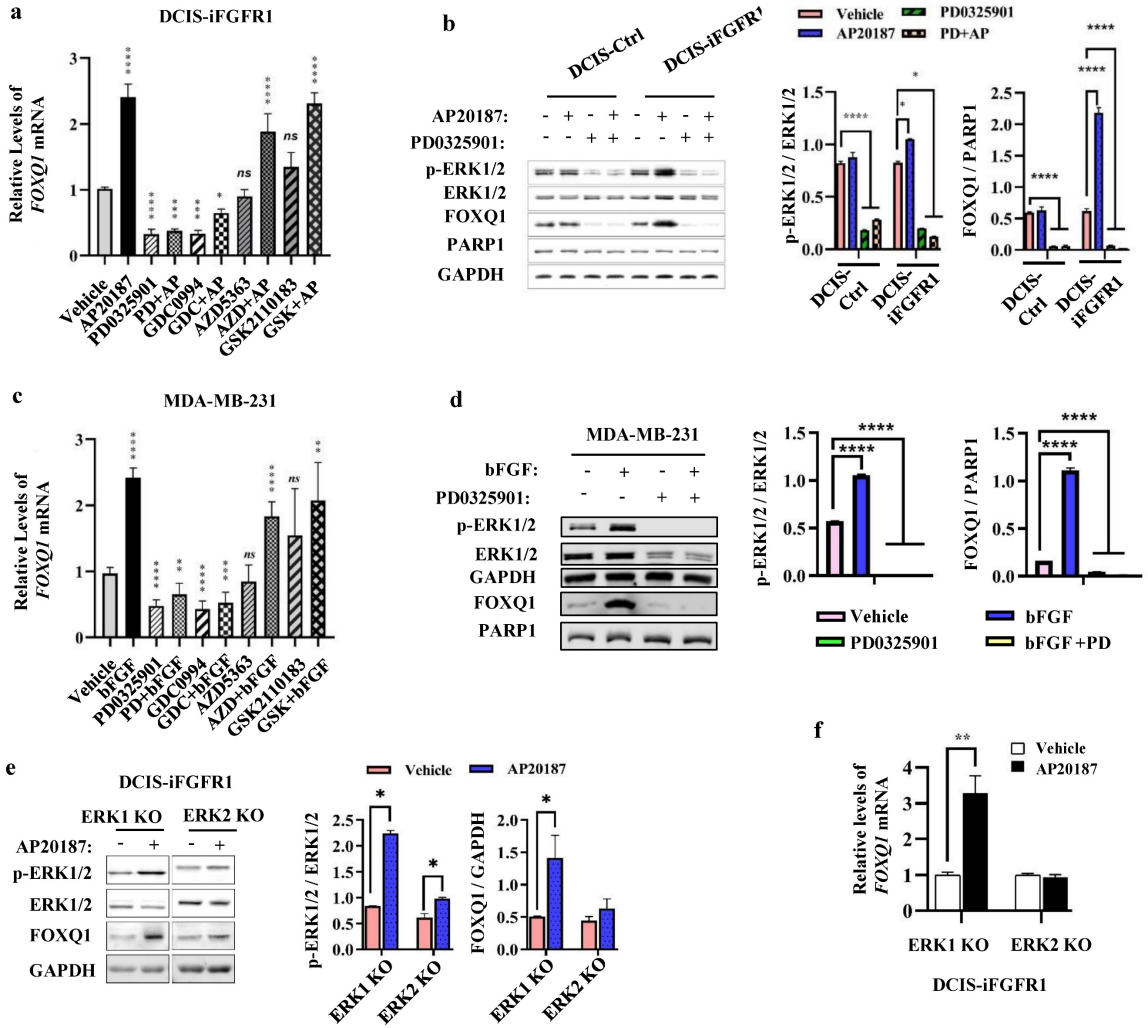
** FGFR1 signaling upregulates FOXQ1 expression mainly through the ERK2-mediated downstream pathway. a.** AP20187-induced FOXQ1 mRNA expression was inhibited by inhibitors of MEK or ERK1/2 in DCIS-iFGFR1 cells. DCIS-iFGFR1 cells were treated with vehicle or AP20187 in combination with 100 nM PD0325901 (a MEK inhibitor), 30 μM GDC0994 (an ERK1/2 inhibitor), 5 μM AZD5363 (an AKT inhibitor), or 2 μM GSK2110183 (an AKT inhibitor) as indicated. The relative expression level of FOXQ1 mRNA was assessed by RT-qPCR, and normalized to the expression level of β-actin mRNA. **b.** The effects of MEK inhibitor PD0325901 on the basal and the AP20187-induced ERK1/2 phosphorylation and FOXQ1 protein expression in DCIS-Ctrl and DCIS-iFGFR1 cells. Cells were treated with vehicle (-), AP20187, and/or PD0325901 as indicated. The indicated proteins were assayed by Western blot (WB). The average ratios of p-ERK1/2 to total ERK1/2, and FOXQ1 to PARP1 are presented in the two bar graphs. **c.** Inhibition of MEK or ERK1/2 blocked bFGF-induced FOXQ1 mRNA expression in MDA-MB-231 cells. Cells were treated with vehicle, bFGF, PD0325901, GDC0994, AZD5363, and/or GSK2110183 as indicated. The relative expression level of FOXQ1 mRNA was measured as described in Panel a. **d.** Inhibition of MEK blocked bFGF-induced FOXQ1 protein expression in MDA-MB-231 cells. Cells were treated with vehicle (-), bFGF, and/or PD0325901 as indicated. The indicated proteins were assayed by WB. The average ratios of p-ERK1/2 to total ERK1/2, and FOXQ1 to PARP1 are presented in the two bar graphs. **e and f.** The effects of ERK1 knockout or ERK2 knockout on the level of phosphorylated ERK2 or ERK1 and the expression levels of FOXQ1 protein and mRNA induced by FGFR1 activation. DCIS-iFGFR1 cells with ERK1 or ERK2 knockout were treated with vehicle or AP20187 (100 nM) for 3 hours. The indicated proteins were assayed by Western blot (Panel e), and the ratios of p-ERK1/2 to total ERK1/2 and FOXQ1 to GAPDH band intensities were presented. The FOXQ1 mRNA level was measured by qPCR and normalized by β-actin mRNA (Panel f). Data in all bar graphs were obtained from 3 independent experiments and presented as mean ± SD. ^*^, ^**^, ^***^, ^****^, and ns, *p* < 0.05, 0.01, 0.001, 0.0001 and not significant, respectively were compared with the appropriate control groups by One-Way ANOVA test (Panels a, b, c, and d) or unpaired Student's t-test (Panel e and f).

FGFR1 activation triggers phosphorylation cascade events to activate MEK-ERK1/2 and PI3K-AKT pathways 14, which promote cancer cell proliferation and survival 41, 42. To determine which downstream pathways are mainly responsible for FGFR1 signaling stimulated FOXQ1 upregulation, DCIS-iFGFR1 cells were treated with vehicle or AP20187 in combination with or without different inhibitors of MEK, ERK, or AKT. Treatment with PD0325901, a MEK inhibitor 43, or GDC0994, an ERK inhibitor 44, effectively inhibited both basal and AP20187-induced FOXQ1 mRNA expression. However, treatment with AZD5363, an AKT inhibitor 45, or GSK2110183, another AKT inhibitor 46, had no significant effects on both basal and AP20187-induced FOXQ1 mRNA expression (Fig. 3a). Accordingly, inhibition of MEK with PD0325901 reduced both basal and AP20187-stimulated phosphorylation of ERK1/2, which also drastically decreased both basal and AP20187-induced expression of FOXQ1 protein (Fig. 3b). Consistent results were also obtained from MDA-MB-231 breast cancer cells with endogenous FGFR1 protein expression, in which the bFGF-induced FOXQ1 mRNA and protein expression can be blocked by MEK/ERK inhibitors but not by AKT inhibitors (Fig. 3c and d). To assess the specific role of ERK1 or ERK2 activation in FGFR1 signaling-promoted FOXQ1 expression, we treated ERK1 knockout or ERK2 knockout DCIS-iFGFR1 cells generated previously 15 with vehicle or AP20187 and assayed FOXQ1 expression. We found that activation of FGFR1 signaling increased ERK2 phosphorylation in ERK1 knockout cells and ERK1 phosphorylation in ERK2 knockout cells. Activation (phosphorylation) of ERK2 in ERK1 knockout cells robustly increased FOXQ1 protein expression, while activation of ERK1 in ERK2 knockout cells did not significantly increase FOXQ1 protein expression (Fig. 3e). In agreement with FOXQ1 protein expression patterns, FOXQ1 mRNA was markedly increased in AP20187-treated ERK1 knockout cells, but not in ERK2 knockout cells (Fig. 3f). These results demonstrate that activation of FGFR1 signaling upregulates FOXQ1 expression via activating the downstream-signaling components MEK and ERK2.

### ERK2 and ERK2-mediated FOXQ1 upregulation are required for FGFR1 signaling-promoted cell growth

**Figure 4:**
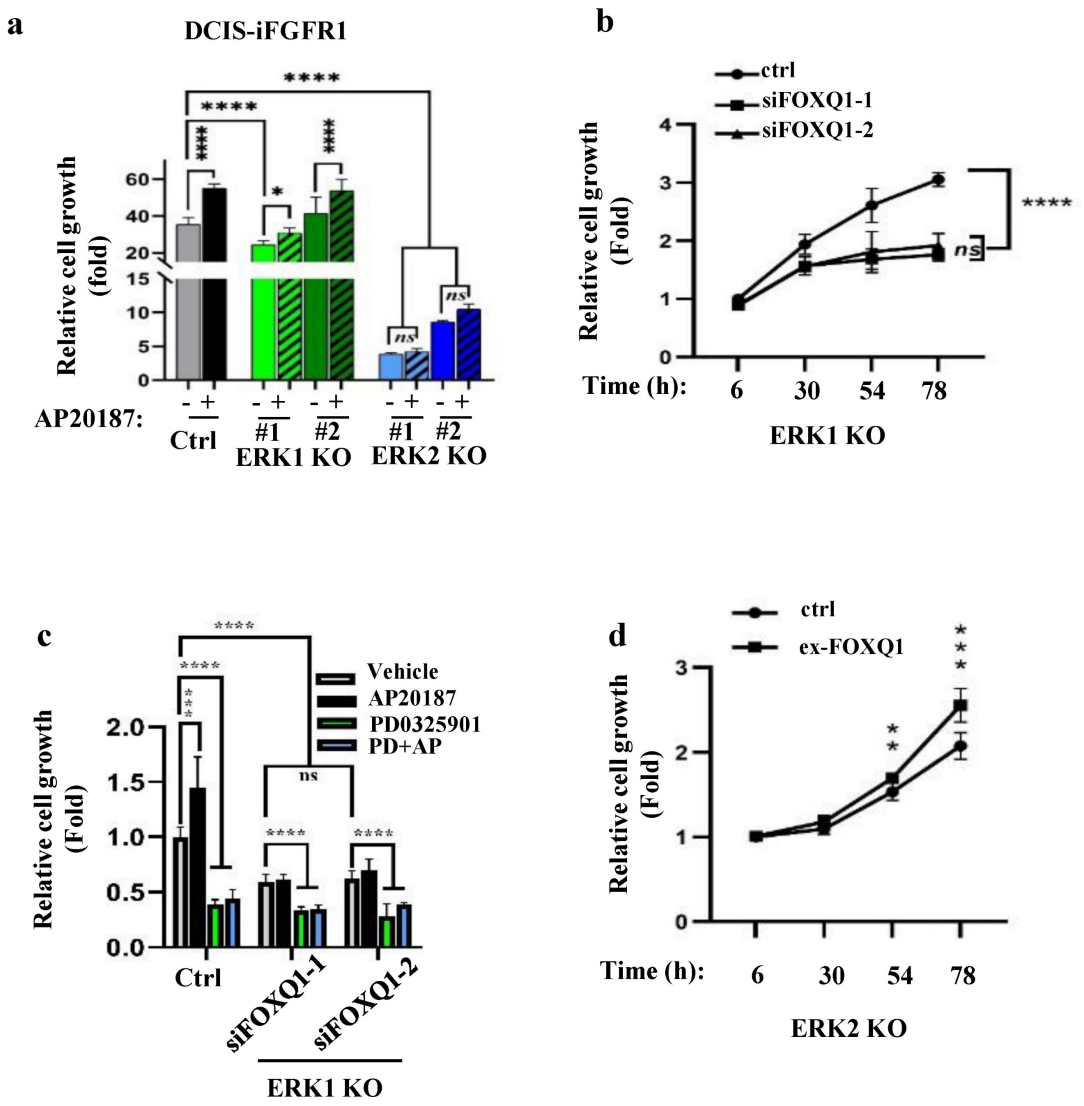
** The FGFR1-signaling pathway depends on ERK2 and FOXQ1 to promote cell growth. a.** Knockout of ERK2 diminished FGFR1 signaling-promoted proliferation of DCIS-iFGFR1 cells. Control, ERK1 knockout (KO) (#1 and #2 lines), and ERK2 KO (#1 and #2 lines) cells derived from DCIS-iFGFR1 cells were treated with vehicle (-) or AP20187 as indicated. Cell viability assay of 6 parallel samples for each group was performed using the Cell Counting Kit-8 (CCK-8). Relative cell growth in fold changes was presented as the average ratio of the OD values at the experimental endpoint (day 3) to that at the experimental start point (day 0). **b.** Knockdown of FOXQ1 in ERK1 knockout DCIS-iFGFR1 cells transfected with siFOXQ1-1 or siFOXQ1-2 siRNA decreased cell proliferation when compared with ERK1 knockout DCIS-iFGFR1 cells transfected with a non-targeting scrambled siRNA (ctrl). Cell proliferation assays were performed at the time points indicated. The data were normalized by setting the value at hour 6 to 1. **c.** Inhibition of MEK/ERK2 or knockdown of FOXQ1 in ERK1 knockout DCIS-iFGFR1 cells diminished AP20187-promoted cell proliferation. ERK1 KO cells were transfected with a non-targeting siRNA (ctrl), siFOXQ1-1 siRNA, or siFOXQ1-2 siRNA. Cells were treated with vehicle, AP20187 (AP), and/or PD0325901 (PD) as indicated. Relative cell growth was assessed after being treated for three days, and presented as normalized data by setting the value of the vehicle-treated group to 1. **d.** Expression of FOXQ1 in ERK2 KO cells increased cell proliferation. ERK2 KO cells were transfected with an empty vector (ctrl) or the FOXQ1-expressing vector (ex-FOXQ1). Relative cell growth was assessed at the time points indicated. Data in all panels were obtained from 6-8 assayed samples and presented as mean ± SD. ^*^, ^**^, ^***^, ^****^, and ns, *p* < 0.05, 0.01, 0.001, 0.0001 and not significant, respectively, which were analyzed by One-Way ANOVA test (Panels a-c) or unpaired Student's t-test (Panel d).

Since FGFR1 signaling upregulates *FOXQ1* gene expression mainly through activating ERK2, we examined how the knockout of ERK2 or ERK1 affects FGFR1 signaling**-**promoted breast cancer cell growth**.** AP20187 treatment significantly accelerated the growth rates of DCIS**-**iFGFR1 cells and two lines of ERK1**-**knockout DCIS**-**iFGFR1 cells**.** However, the two lines of ERK2**-**knockout DCIS**-**iFGFR1 cells grew much slower than DCIS**-**iFGFR1 control cells and ERK1**-**knockout DCIS**-**iFGFR1 cells in both the absence and presence of AP20187 **(**Fig**.** 4a**).** Since activation of FGFR1 signaling in ERK1 knockout cells still upregulated FOXQ1, we transfected ERK1 knockout cells with a non**-**targeting control siRNA and two different FOXQ1 mRNA**-**targeting siRNAs and compared their growth rates**.** We found that cells transfected with the targeting siRNAs grew much slower than the cells transfected with the non**-**targeting siRNA, suggesting that FOXQ1 plays a role in supporting ERK1 knockout cell proliferation **(**Fig**.** 4b**).** Furthermore, the proliferation of ERK1 knockout DCIS**-**iFGFR1 cells without FOXQ1 knockdown was inhibited more than two folds by inhibiting ERK2 with PD0325901**.** The proliferation of the same cells stimulated by AP20187 was inhibited more than three folds by PD032901**.** Moreover, the knockdown of FOXQ1 in ERK1 knockout DCIS**-**iFGFR1 cells abolished the AP20187**-**stimulated cell proliferation, and PD032901 equally inhibited ERK1 knockout DCIS**-**iFGFR1 cells with FOXQ1 knockdown in either absence or presence of AP20187 treatment **(**Fig**.** 4c**).** Finally, expression of FOXQ1 in ERK2 knockout DCIS**-**iFGFR1 cells significantly improved cell proliferation **(**Fig**.** 4d**).** Collectively, these results demonstrate that FGFR1 signaling depends on ERK2 and ERK2**-**mediated FOXQ1 upregulation to promote breast cancer cell proliferation**.**

### c*-*FOS binds the *FOXQ1* gene promoter to activate its transcriptional activity

**Figure 5:**
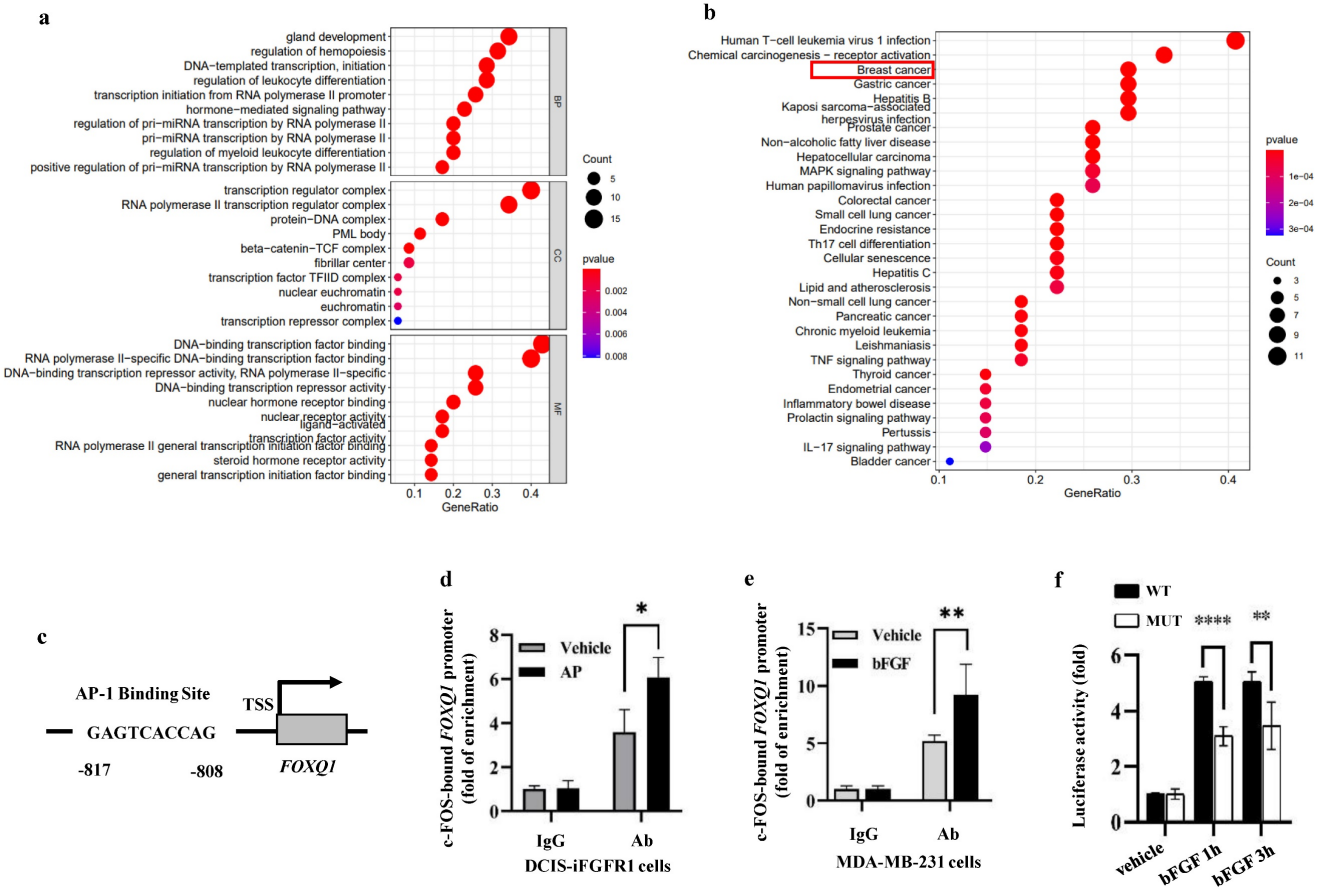
** c-FOS regulates the FOXQ1 promoter activity. a and b.** Gene Ontology (GO) enrichment analysis (Panel a) and KEGG pathway analysis (Panel b) of the 55 transcription factors predicted by the PROMO database. **c.** A schematic diagram of the c-FOS-binding site in the human *FOXQ1* gene promoter. **d and e.** ChIP-qPCR assays. Cross-linked protein-chromatin complexes were prepared from DCIS-iFGFR1 cells treated with vehicle or AP20187 or MDA-MB-231 cells treated with vehicle or bFGF. ChIP was performed using a c-FOS antibody. The non-immune IgG was used as a negative control. qPCR was performed to measure the FOXQ1 promoter sequence containing the c-FOS-binding site shown in Panel c. **f.** Dual luciferase assays. Relative luciferase activities of the FOXQ1-WT and FOXQ1-MUT reporters were determined in HEK293 cells treated with vehicle or bFGF as indicated. The quantitative data in Panels d-f were obtained from 3 independent assays and presented as mean ± SD. ^*^, ^**^, and ^****^, *p* < 0.05, 0.01, and 0.0001, respectively, compared by unpaired Student's t-test.

In order to identify a transcription factor that mediates FGFR1**/**ERK2**-**promoted FOXQ1 upregulation, we analyzed the 5**'** regulatory sequence of the *FOXQ1* gene by bioinformatics algorithm with UCSC and PROMO databases to look for binding motifs of transcription factors regulated by the ERK signaling pathway**.** When the dissimilarity was 5**%**, the analysis predicted 55 transcription factors, including GR**-**α, PR**-**α, PAX5, GATA**-**1, c**-**FOS, c**-**JUN, AP**-**1, and P53 that are related to growth, proliferation, differentiation, and apoptosis **(**Supplemental Table S2**).** Gene Ontology **(**GO**)** enrichment analysis showed that these transcription factors**'** biological processes **(**BP**)** were mainly related to gland development, regulation of hemopoiesis, DNA**-**templated transcription and initiation, etc**. (**Fig 5a**).** KEGG pathway analysis revealed that these 55 transcription factors were enriched in human T**-**cell leukemia virus 1 infection, chemical carcinogenesis**-**receptor activation, breast and other cancers, and MAPK signaling pathways**.** Eight transcription factors, TP53, JUN, FOS, ESR1, RB1, E2F1, TCF7L2, and LEF1, are involved in the breast cancer signaling pathway **(**Fig**.** 5b**).**

Since c**-**FOS is a well**-**established early response gene upregulated by the MEK**/**ERK signaling pathway **47**, we studied how c**-**FOS regulates *FOXQ1* gene expression**.** A c**-**FOS binding motif was predicted in the *FOXQ1* gene promoter region from **-**817 to **-**808 bp to the transcription start site **(**TSS**) (**Fig**.** 5c**).** We performed chromatin immunoprecipitation **(**ChIP**)** assays to examine whether c**-**FOS could bind to the *FOXQ1* gene promoter**.** DCIS**-**iFGFR1 cells were treated with vehicle as a control, or with AP20187 to activate FGFR1 signaling**.** The ChIP assay was performed with IgG as a control and with a c**-**FOS antibody**.** c**-**FOS was detected to be associated with the *FOXQ1* gene promoter at a moderate level in vehicle**-**treated cells**.** This association is significantly enhanced in AP20187**-**treated cells **(**Fig**.** 5d**).** Similar results were also obtained from MDA**-**MB**-**231 breast cancer cells with endogenous *FGFR1* expression, where c**-**FOS recruitment to the *FOXQ1* gene promoter was increased about two folds upon bFGF treatment **(**Fig**.** 5e**).** These results indicate that c**-**FOS binds the *FOXQ1* promoter and this association is increased significantly upon the activation of the FGFR1 signaling pathway**.**

To test whether c**-**FOS regulates the activity of the *FOXQ1* gene promoter, we constructed a pGL3 luciferase reporter, FOXQ1**-**WT, driven by the *FOXQ1* gene promoter sequence from bp **-**1000 to the 1^st^ bp of exon 1 that included the predicted c**-**FOS binding motif**.** We used HEK293 cells for the reporter**-**based transcription assay because of their high transfection efficiency**.** The pGL3**-**basic control vector expressed nearly no luciferase activity in HEK293 cells treated with vehicle, TPA, or TPA and T**-**5224**.** TPA is a stimulator of the MEK**/**ERK pathway and the c**-**FOS activity **48**, which was used to activate c**-**FOS in HEK293 cells**.** T**-**5224 is a c**-**FOS**/**AP**-**1 inhibitor **49**, which was used to inhibit c**-**FOS activity**.** Interestingly, the FOXQ1**-**WT reporter expressed a moderate level of luciferase reporter activity in the vehicle**-**treated cells; TPA treatment further increased the luciferase reporter activity more than two folds, and this TPA**-**induced reporter activity could be inhibited by T**-**5224 treatment **(**Supplemental Fig. S2**).** These results suggest that c**-**FOS is one of the major transcription factors that strongly activate the *FOXQ1* gene promoter**.** To further define the specific role of c**-**FOS in regulating the *FOXQ1* gene promoter, we constructed another luciferase reporter, designated as FOXQ1**-**MUT, which had a deletion of the predicted c**-**FOS**-**binding motif in the same *FOXQ1* gene promoter sequence**.** In the transfected HEK293 cells, bFGF treatment dramatically increased the luciferase activity of the FOXQ1**-**WT reporter, while deletion of the c**-**FOS binding motif significantly decreased the luciferase activity of FOXQ1**-**MUT reporter **(**Fig**.** 5f**).** These results indicate that the transcriptional activity of the *FOXQ1* gene promoter is directly upregulated by c**-**FOS upon bFGF treatment**.**

### The FGFR1-MEK-ERK2 signaling pathway upregulates c-FOS to promote *FOXQ1* gene expression

**Figure 6:**
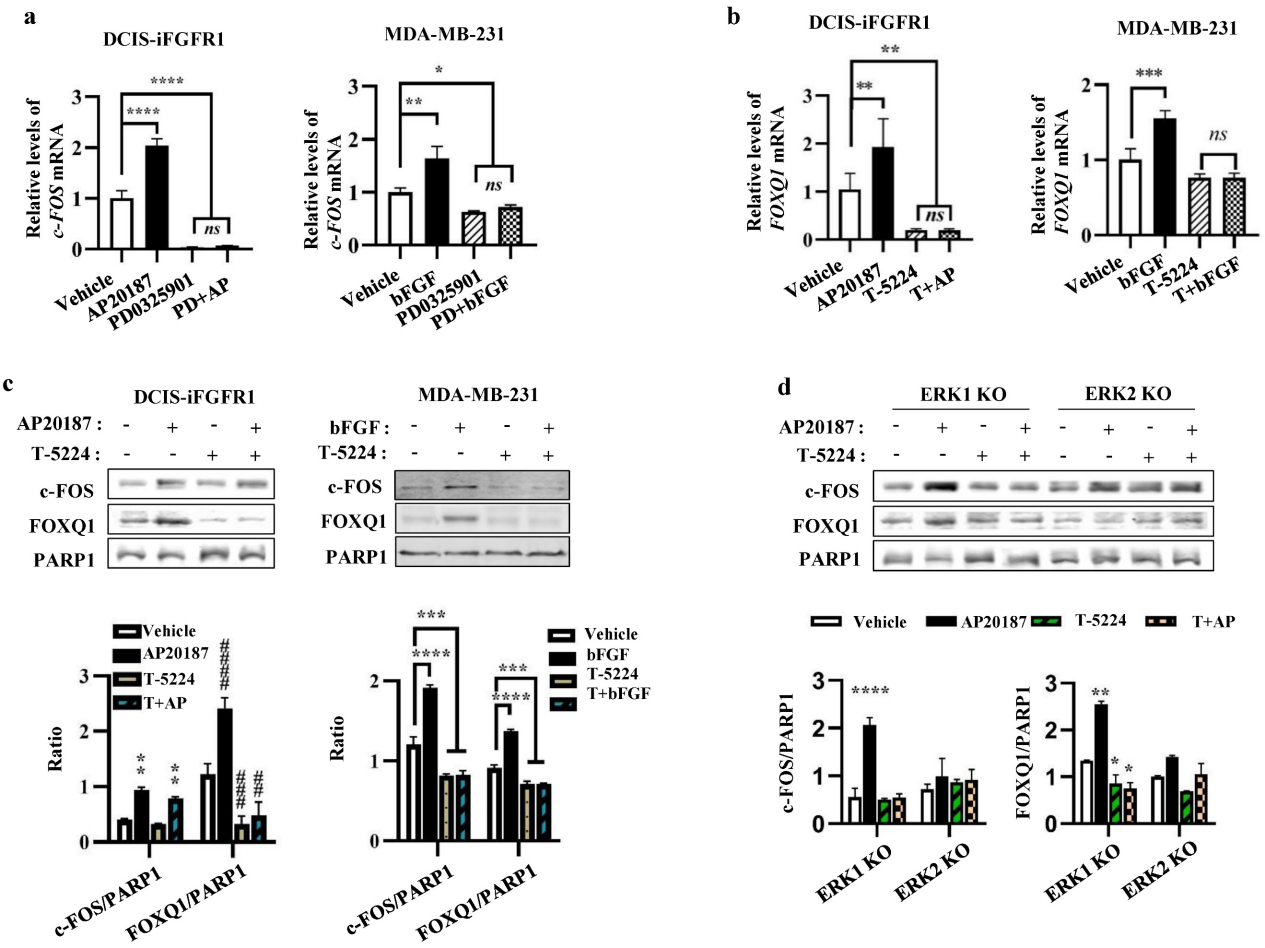
** c-FOS plays an essential role in the FGFR1-MEK-ERK2 signaling pathway-promoted FOXQ1 expression. a.** Inhibition of MEK with PD0325901, which prevents ERK1/2 phosphorylation, repressed basal and AP20187- or bFGF-induced c-FOS mRNA expression in DCIS-iFGFR1 and MDA-MB-231 cells, respectively. Cells were treated with vehicle, AP20187 (AP), bFGF, and/or PD0325901 (PD) as indicated. c-FOS mRNA levels were assayed by qPCR and normalized to β-actin mRNA levels. **b.** Inhibition of c-FOS activity repressed AP20187- and bFGF-induced FOXQ1 mRNA expression in DCIS-iFGFR1 and MDA-MB-231 cells, respectively. Cells were treated with vehicle, AP20187 (AP), bFGF, and/or T-5224 as indicated. FOXQ1 mRNA levels were assayed by qPCR and normalized to β-actin mRNA levels. **c.** Inhibition of c-FOS repressed AP20187- or bFGF-induced FOXQ1 protein expression in DCIS-iFGFR1 and MDA-MB-231 cells, respectively. Cells were treated with vehicle, AP20187, and/or T-5224 as indicated. The levels of c-FOS and FOXQ1 proteins were measured by Western blot (WB), and normalized to the levels of PARP1, a nuclear protein. **d.** c-FOS activity is required for AP20187-induced FOXQ1 expression in ERK1 knockout DCIS-iFGFR1 cells. ERK1-knockout (KO) and ERK2-KO DCIS-iFGFR1 cells were treated with vehicle, AP20187, and/or T-5224 as indicated. The levels of c-FOS and FOXQ1 proteins were assayed by WB and normalized to the levels of PARP1 protein. In all bar graphs, quantitative data were obtained from 3 independent assays and presented as mean ± standard deviation (SD). ^*^, ^** (##)^, ^*** (###)^, and ^**** (####)^, *p* < 0.05, 0.01, 0.001, and 0.0001, respectively, which were compared by the One-Way ANOVA test.

As expected, the mRNA expression of c**-**FOS, an early response gene of the MEK**/**ERK signaling pathway **47**, was upregulated by AP20187**-** or bFGF**-**activated FGFR1 signaling in DCIS**-**iFGFR1 and MDA**-**MB**-**231 cells, respectively, and inhibited by PD0325901, a MEK inhibitor that also prevents ERK1**/**2 activation, in both types of cells **(**Fig**.** 6a**).** Importantly, the increased expression of c**-**FOS mRNA induced by the activation of FGFR1 signaling in these cells was associated with the increased expression of FOXQ1 mRNA, and the treatment with T**-**5224, a c**-**FOS inhibitor, diminished FOXQ1 mRNA expression promoted by the activated FGFR1 signaling pathway **(**Fig**.** 6b**).** The c**-**FOS protein was also increased in AP20187**-**treated DCIS**-**iFGFR1 cells and bFGF**-**treated MDA**-**MB**-**231 cells**.** This increase was also associated with the increase in FOXQ1 protein expression**.** Inhibition of c**-**FOS with T**-**5224 abolished FOXQ1 protein expression increased by the activated FGFR1 signaling **(**Fig**.** 6c**).** In ERK1 knockout DCIS**-**iFGFR1 cells, activation of FGFR1 signaling by AP20187 treatment still upregulated both c**-**FOS and FOXQ1 proteins, and these upregulations were inhibited by T**-**5224**.** However, in ERK2 knockout DCIS**-**iFGFR1 cells, activation of FGFR1 signaling was unable to increase c**-**FOS and FOXQ1 protein expression, and T**-**5224 did not show any significant effect on c**-**FOS and FOXQ1 protein expression in ERK2 knockout cells **(**Fig**.** 6d**).** These results suggest that the activation of the bFGF**-**FGFR1**-**MEK**-**ERK2 signaling pathway upregulates c**-**FOS expression, which in turn promotes FOXQ1 expression**.** In addition, we also noticed that T**-**5244 treatment did not change the level of c**-**FOS protein in DCIS**-**iFGFR1 cells **(**Fig**.** 6c, bars 1 vs**.** 3, and bars 2 vs**.** 4 in the left bar graph**)**, but it reduced the c**-**FOS protein in MDA**-**MD**-**231 cells **(**Fig**.** 6c, bars 1 vs**.** 3, and bars 2 vs**.** 4 in the right bar graph**)**, and ERK1 knockout cells **(**Fig**.** 6d, bars 1 vs**.** 3, and bars 2 vs**.** 4 in the left bar graph**).** What caused these different effects of the c**-**FOS inhibitor T**-**5244 on c**-**FOS protein levels in different cell lines were still unknown**.**

## Discussion

Both the overactivated FGFR1 signaling and the overexpressed FOXQ1 are oncogenic factors that promote the proliferation, invasiveness, and progression of cancer cells **3-5, 21-26.** However, the regulatory and functional relationships between FGFR1 signaling and FOXQ1 overexpression are unknown**.** In this study, we demonstrated that activation of the FGFR1 signaling robustly upregulated FOXQ1 mRNA and protein expression in breast cancer cells**.** Knockdown of *FOXQ1* gene expression blocked the FGFR1 signaling**-**promoted breast cancer cell proliferation and colony formation in culture, and inhibited the FGFR1 signaling**-**accelerated growth in xenograft tumors derived from breast cancer cells in mice**.** These findings indicate that FOXQ1 protein plays an essential role in the FGFR1 signaling**-**promoted breast cancer cell growth**.** After identifying this important phenotype, we further defined the molecular mechanism responsible for the FGFR1 signaling to upregulate *FOXQ1* expression**.**

The RAS**-**RAF**-**MEK**-**ERK1**/**2 signaling pathway is a major FGFR1**-**activated downstream pathway**.** The activity of this pathway is increased in about one**-**third of all human cancers **50.** Previous studies have demonstrated that activation of ERK1**/**2 by FGFR1 signaling is crucial to FGFR1 signaling**-**regulated cell proliferation and differentiation **51.** Nevertheless, the specific contribution of ERK1 or ERK2 to these cellular processes has not been fully defined**.** Although many functions of ERK1**/**2 may be redundant, each may possess certain unique functions, as evidenced by the survival phenotype of *ERK1* knockout mice and the lethal phenotype of *ERK2* knockout mice **52, 53.** In NIH 3T3 cells, ERK1 and ERK2 also showed some different effects on RAS**-**dependent cell signaling **54.** In this study, we found that activation of the FGFR1 signaling enhances the phosphorylation of both ERK1 and ERK2, and inhibition of MEK to prevent the activation of both ERK1 and ERK2 blocked the FGFR1 signaling**-**induced FOXQ1 upregulation and breast cancer cell proliferation**.** However, inhibition of the AKT activity did not significantly affect FOXQ1 expression, suggesting that the FGFR1**-**PI3K**-**AKT pathway is not essential for the FGFR1 signaling**-**induced FOXQ1 expression**.** Interestingly, knockout of ERK2, but not ERK1, specifically suppressed the FGFR1 signaling**-**induced FOXQ1 expression and breast cancer cell proliferation**.** Moreover, inhibition of ERK2 in ERK1 knockout cells suppressed the FGFR1 signaling**-**promoted FOXQ1 expression and cell proliferation, while restored FOXQ1 expression in ERK2 knockout cells increased the proliferation rate of these cells**.** Together, these findings support that activation of the FGFR1 signaling mainly upregulates FOXQ1 expression and breast cancer cell proliferation through activating ERK2, but not ERK1, in the FGFR1**-**MEK**-**ERK2 signaling pathway**.**

It is known that activated ERK1**/**2 can result in upregulation and**/**or activation of multiple transcription factors such as c**-**FOS, ETS, and ELK **50.** The* c****-****Fos* gene is the human homolog of the retroviral oncogene *v****-****fos* and is considered a protooncogene **55.** Because *c****-****FOS* expression can be rapidly induced by growth factor stimulation within 15 minutes, it is referred to as one of the early growth response genes **56.** The *c****-****Fos* gene encodes four proteins**:** c**-**FOS, FOSB, FOSL1, and FOSL2**.** c**-**FOS protein heterodimerizes with one of the JUN family members to form activator protein**-**1 **(**AP**-**1**)** that binds DNA to regulate transcription of their target genes important for cell proliferation, differentiation, apoptosis, migration, and transformation **57, 58.** In this study, we demonstrated that the human *FOXQ1* gene promoter contains a functional AP**-**1 binding motif that is associated with c**-**FOS and required for bFGF**-**stimulated transcriptional activity of the *FOXQ1* gene promoter in breast cancer cells**.** We also demonstrated that activation of the FGFR1 signaling induced c**-**FOS upregulation in a MEK and ERK2 function**-**dependent manner and FOXQ1 upregulation in an AP**-**1 function**-**dependent manner**.** These results indicate that activation of the FGFR1**-**MEK**-**ERK2 signaling pathway causes c**-**FOS upregulation, which in turn binds to the *FOXQ1* promoter to upregulate *FOXQ1* expression.

Compared with c**-**FOS, FOXQ1 is an understudied protooncogenic transcription factor for its upstream regulators, downstream direct target genes, and mechanism**-**based roles in breast cancer **29-31.** Previous studies have shown that FOXQ1 could mediate the roles of TGF**-**β and Wnt signaling pathways in inducing the epithelial**-**to**-**mesenchymal transition **(**EMT**)** in cancer cells **59, 60.** FOXQ1 regulates the expression levels of cell cycle regulators such as cyclin D1, cyclin E, CDK4, p27^Kip1^, and p21^Cip1^ to maintain and promote cell proliferation **61.** Our study demonstrated that activation of the FGFR1**-**MEK**-**ERK2**-**c**-**FOS regulatory axis robustly upregulates FOXQ1**.** In contrast, the knockdown of FOXQ1 inhibited the role of FGFR1 signaling in the stimulation of breast cancer cell proliferation and colony formation in culture, as well as the growth of human breast cancer cell**-**derived xenografts in mice**.** These findings indicate that FOXQ1 plays a key role in FGFR1 signaling**-**stimulated breast cancer growth.

**Figure 7:**
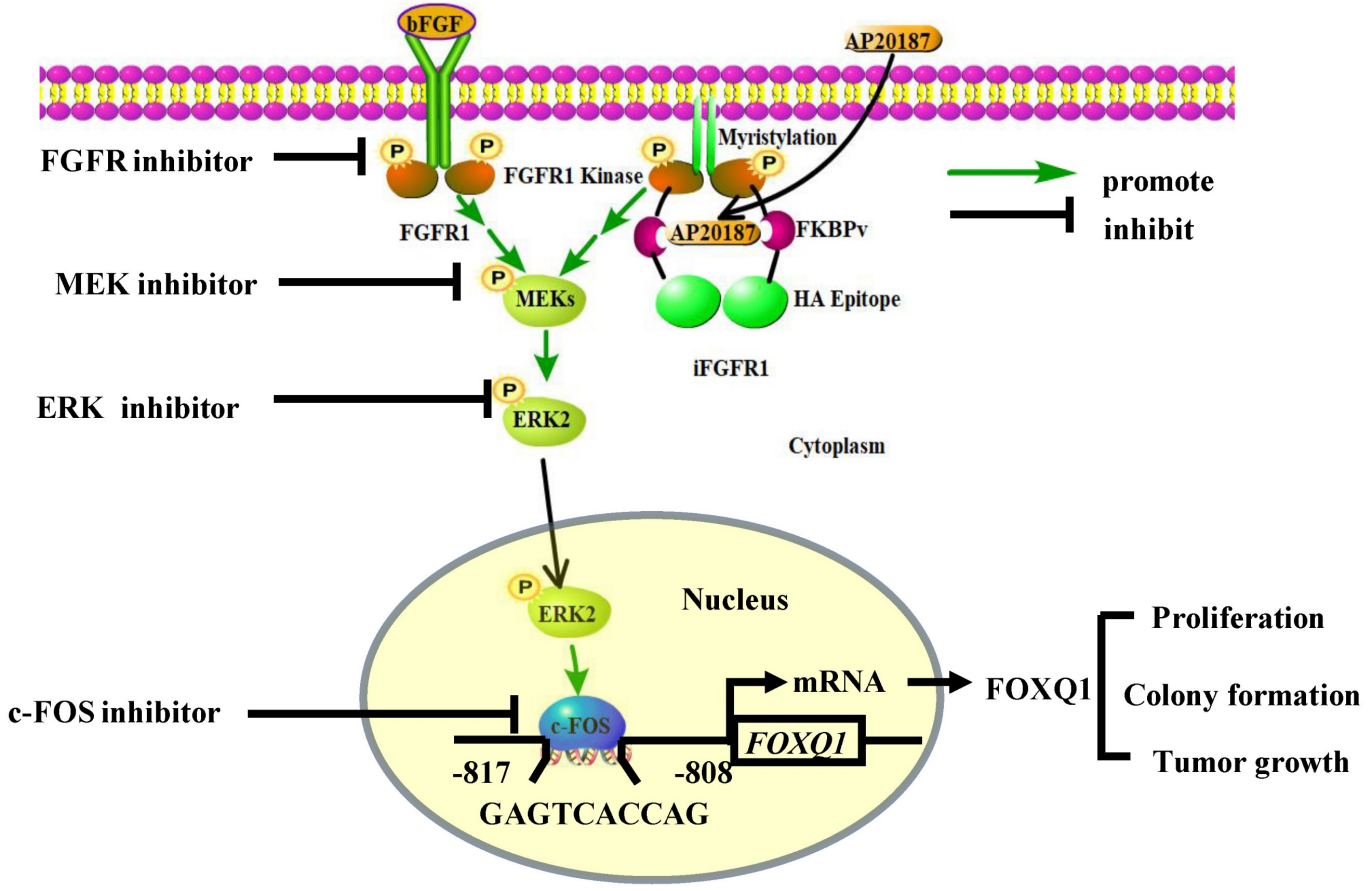
** A schematic diagram of the bFGF-FGFR1-ERK2-c-FOS-FOXQ1 gene regulatory axis.** The FGFR1/iFGFR1 signaling can be activated by bFGF or AP20187, which activates MEK and ERK2, resulting in c-FOS upregulation. c-FOS binds to the FOXQ1 promoter to upregulate FOXQ1 expression, which in turn promotes cancer cell proliferation, colony formation, and tumor growth.

In summary, we demonstrated that activation of the FGFR1 signaling robustly upregulates FOXQ1 expression through activating FGFR1**-**MEK**-**ERK2 to upregulate c**-**FOS and c**-**FOS**-**enhanced *FOXQ1* gene promoter activity, and FOXQ1 plays an essential role in mediating the FGFR1 signaling**-**promoted breast cancer cell proliferation, colony formation and tumor growth **(**Fig**.** 7**).** Therefore, the growth of breast cancer cells driven by the deregulated FGFR1 signaling can be suppressed by targeting ERK2 and FOXQ1 once these cancer cells become resistant to FGFR1 inhibitors.

## Supplementary Material

Supplementary figures and tables.

## References

[1] Sung H, Ferlay J, Siegel RL, Laversanne M, Soerjomataram I, Jemal A (2021). Global Cancer Statistics 2020: GLOBOCAN Estimates of Incidence and Mortality Worldwide for 36 Cancers in 185 Countries. CA Cancer J Clin.

[2] Helsten T, Elkin S, Arthur E, Tomson BN, Carter J, Kurzrock R (2016). The FGFR Landscape in Cancer: Analysis of 4,853 Tumors by Next-Generation Sequencing. Clin Cancer Res.

[3] Babina IS, Turner NC (2017). Advances and challenges in targeting FGFR signalling in cancer. Nature reviews Cancer.

[4] Tarkkonen KM, Nilsson EM, Kahkonen TE, Dey JH, Heikkila JE, Tuomela JM (2012). Differential roles of fibroblast growth factor receptors (FGFR) 1, 2 and 3 in the regulation of S115 breast cancer cell growth. PloS one.

[5] Xian W, Pappas L, Pandya D, Selfors LM, Derksen PW, de Bruin M (2009). Fibroblast growth factor receptor 1-transformed mammary epithelial cells are dependent on RSK activity for growth and survival. Cancer Res.

[6] Turner N, Pearson A, Sharpe R, Lambros M, Geyer F, Lopez-Garcia MA (2010). FGFR1 amplification drives endocrine therapy resistance and is a therapeutic target in breast cancer. Cancer Res.

[7] Kim B, Wang S, Lee JM, Jeong Y, Ahn T, Son DS (2015). Synthetic lethal screening reveals FGFR as one of the combinatorial targets to overcome resistance to Met-targeted therapy. Oncogene.

[8] Anreddy N, Patel A, Sodani K, Kathawala RJ, Chen EP, Wurpel JN (2014). PD173074, a selective FGFR inhibitor, reverses MRP7 (ABCC10)-mediated MDR. Acta Pharm Sin B.

[9] Terai H, Soejima K, Yasuda H, Nakayama S, Hamamoto J, Arai D (2013). Activation of the FGF2-FGFR1 autocrine pathway: a novel mechanism of acquired resistance to gefitinib in NSCLC. Mol Cancer Res.

[10] Gyanchandani R, Ortega Alves MV, Myers JN, Kim S (2013). A proangiogenic signature is revealed in FGF-mediated bevacizumab-resistant head and neck squamous cell carcinoma. Mol Cancer Res.

[11] Navid S, Fan C, P OF-V, Generali D, Li Y (2020). The Fibroblast Growth Factor Receptors in Breast Cancer: from Oncogenesis to Better Treatments. International journal of molecular sciences.

[12] Xie Y, Su N, Yang J, Tan Q, Huang S, Jin M (2020). FGF/FGFR signaling in health and disease. Signal Transduct Target Ther.

[13] Huang Z, Marsiglia WM, Basu Roy U, Rahimi N, Ilghari D, Wang H (2016). Two FGF Receptor Kinase Molecules Act in Concert to Recruit and Transphosphorylate Phospholipase Cgamma. Mol Cell.

[14] Turner N, Grose R (2010). Fibroblast growth factor signalling: from development to cancer. Nature reviews Cancer.

[15] Yang M, Yu X, Li X, Luo B, Yang W, Lin Y (2018). TNFAIP3 is required for FGFR1 activation-promoted proliferation and tumorigenesis of premalignant DCIS.COM human mammary epithelial cells. Breast cancer research: BCR.

[16] Ji W, Yu Y, Li Z, Wang G, Li F, Xia W (2016). FGFR1 promotes the stem cell-like phenotype of FGFR1-amplified non-small cell lung cancer cells through the Hedgehog pathway. Oncotarget.

[17] Wang K, Ji W, Yu Y, Li Z, Niu X, Xia W (2018). FGFR1-ERK1/2-SOX2 axis promotes cell proliferation, epithelial-mesenchymal transition, and metastasis in FGFR1-amplified lung cancer. Oncogene.

[18] Lu Y, Liu Y, Oeck S, Zhang GJ, Schramm A, Glazer PM (2020). Hypoxia Induces Resistance to EGFR Inhibitors in Lung Cancer Cells via Upregulation of FGFR1 and the MAPK Pathway. Cancer research.

[19] Reed JR, Stone MD, Beadnell TC, Ryu Y, Griffin TJ, Schwertfeger KL (2012). Fibroblast growth factor receptor 1 activation in mammary tumor cells promotes macrophage recruitment in a CX3CL1-dependent manner. PloS one.

[20] Li Y, Zhang Y, Yao Z, Li S, Yin Z, Xu M (2016). Forkhead box Q1: A key player in the pathogenesis of tumors (Review). International journal of oncology.

[21] Zhang J, Yang Y, Yang T, Yuan S, Wang R, Pan Z (2015). Double-negative feedback loop between microRNA-422a and forkhead box (FOX)G1/Q1/E1 regulates hepatocellular carcinoma tumor growth and metastasis. Hepatology (Baltimore, Md).

[22] Sun HT, Cheng SX, Tu Y, Li XH, Zhang S (2013). FoxQ1 promotes glioma cells proliferation and migration by regulating NRXN3 expression. PloS one.

[23] Kaneda H, Arao T, Tanaka K, Tamura D, Aomatsu K, Kudo K (2010). FOXQ1 is overexpressed in colorectal cancer and enhances tumorigenicity and tumor growth. Cancer research.

[24] Vishnubalaji R, Hamam R, Yue S, Al-Obeed O, Kassem M, Liu FF (2016). MicroRNA-320 suppresses colorectal cancer by targeting SOX4, FOXM1, and FOXQ1. Oncotarget.

[25] Zhang H, Meng F, Liu G, Zhang B, Zhu J, Wu F (2011). Forkhead transcription factor foxq1 promotes epithelial-mesenchymal transition and breast cancer metastasis. Cancer research.

[26] Feng J, Zhang X, Zhu H, Wang X, Ni S, Huang J (2012). FoxQ1 overexpression influences poor prognosis in non-small cell lung cancer, associates with the phenomenon of EMT. PloS one.

[27] Xia L, Huang W, Tian D, Zhang L, Qi X, Chen Z (2014). Forkhead box Q1 promotes hepatocellular carcinoma metastasis by transactivating ZEB2 and VersicanV1 expression. Hepatology (Baltimore, Md).

[28] Bao B, Azmi AS, Aboukameel A, Ahmad A, Bolling-Fischer A, Sethi S (2014). Pancreatic cancer stem-like cells display aggressive behavior mediated via activation of FoxQ1. The Journal of biological chemistry.

[29] Cui Z, Zhao Y (2019). microRNA-342-3p targets FOXQ1 to suppress the aggressive phenotype of nasopharyngeal carcinoma cells. BMC cancer.

[30] Wu X, Gardashova G, Lan L, Han S, Zhong C, Marquez RT (2020). Targeting the interaction between RNA-binding protein HuR and FOXQ1 suppresses breast cancer invasion and metastasis. Commun Biol.

[31] Tang H, Zhang J, Guo Q (2018). Research progress on the regulation of tumor initiation and development by the forkhead box Q1 gene. Journal of cancer research and therapeutics.

[32] Miller FR, Santner SJ, Tait L, Dawson PJ (2000). MCF10DCIS.com xenograft model of human comedo ductal carcinoma in situ. J Natl Cancer Inst.

[33] Freeman KW, Gangula RD, Welm BE, Ozen M, Foster BA, Rosen JM (2003). Conditional activation of fibroblast growth factor receptor (FGFR) 1, but not FGFR2, in prostate cancer cells leads to increased osteopontin induction, extracellular signal-regulated kinase activation, and in vivo proliferation. Cancer Res.

[34] Messeguer X, Escudero R, Farré D, Núñez O, Martínez J, Albà MM (2002). PROMO: detection of known transcription regulatory elements using species-tailored searches. Bioinformatics.

[35] Farré D, Roset R, Huerta M, Adsuara JE, Roselló L, Albà MM (2003). Identification of patterns in biological sequences at the ALGGEN server: PROMO and MALGEN. Nucleic Acids Res.

[36] Wang W, He S, Ji J, Huang J, Zhang S, Zhang Y (2013). The prognostic significance of FOXQ1 oncogene overexpression in human hepatocellular carcinoma. Pathology, research and practice.

[37] Liang SH, Yan XZ, Wang BL, Jin HF, Yao LP, Li YN (2013). Increased expression of FOXQ1 is a prognostic marker for patients with gastric cancer. Tumour biology: the journal of the International Society for Oncodevelopmental Biology and Medicine.

[38] Cheng CL, Thike AA, Tan SY, Chua PJ, Bay BH, Tan PH (2015). Expression of FGFR1 is an independent prognostic factor in triple-negative breast cancer. Breast Cancer Res Treat.

[39] Gavine PR, Mooney L, Kilgour E, Thomas AP, Al-Kadhimi K, Beck S (2012). AZD4547: an orally bioavailable, potent, and selective inhibitor of the fibroblast growth factor receptor tyrosine kinase family. Cancer research.

[40] Zhao G, Li WY, Chen D, Henry JR, Li HY, Chen Z (2011). A novel, selective inhibitor of fibroblast growth factor receptors that shows a potent broad spectrum of antitumor activity in several tumor xenograft models. Mol Cancer Ther.

[41] Engelman JA (2009). Targeting PI3K signalling in cancer: opportunities, challenges and limitations. Nature reviews Cancer.

[42] Zhong H, Wu H, Bai H, Wang M, Wen J, Gong J (2019). Panax notoginseng saponins promote liver regeneration through activation of the PI3K/AKT/mTOR cell proliferation pathway and upregulation of the AKT/Bad cell survival pathway in mice. BMC Complementary and Alternative Medicine.

[43] Henderson YC, Chen Y, Frederick MJ, Lai SY, Clayman GL (2010). MEK inhibitor PD0325901 significantly reduces the growth of papillary thyroid carcinoma cells in vitro and in vivo. Mol Cancer Ther.

[44] Blake JF, Burkard M, Chan J, Chen H, Chou KJ, Diaz D (2016). Discovery of (S)-1-(1-(4-Chloro-3-fluorophenyl)-2-hydroxyethyl)-4-(2-((1-methyl-1H-pyrazol-5-yl)amino)pyrimidin-4-yl)pyridin-2(1H)-one (GDC-0994), an Extracellular Signal-Regulated Kinase 1/2 (ERK1/2) Inhibitor in Early Clinical Development. J Med Chem.

[45] Addie M, Ballard P, Buttar D, Crafter C, Currie G, Davies BR (2013). Discovery of 4-amino-N-[(1S)-1-(4-chlorophenyl)-3-hydroxypropyl]-1-(7H-pyrrolo[2,3-d]pyrimidin-4-yl)piperidine-4-carboxamide (AZD5363), an orally bioavailable, potent inhibitor of Akt kinases. J Med Chem.

[46] Dumble M, Crouthamel MC, Zhang SY, Schaber M, Levy D, Robell K (2014). Discovery of novel AKT inhibitors with enhanced anti-tumor effects in combination with the MEK inhibitor. PloS one.

[47] Gilley R, March HN, Cook SJ (2009). ERK1/2, but not ERK5, is necessary and sufficient for phosphorylation and activation of c-Fos. Cell Signal.

[48] Xu X, Prough RA, Samuelson DJ (2015). Differential 12-O-Tetradecanoylphorbol-13-acetate-induced activation of rat mammary carcinoma susceptibility Fbxo10 variant promoters via a PKC-AP1 pathway. Mol Carcinog.

[49] Ishida M, Ueki M, Morishita J, Ueno M, Shiozawa S, Maekawa N (2015). T-5224, a selective inhibitor of c-Fos/activator protein-1, improves survival by inhibiting serum high mobility group box-1 in lethal lipopolysaccharide-induced acute kidney injury model. Journal of Intensive Care.

[50] Roskoski R (2012). ERK1/2 MAP kinases: Structure, function, and regulation. Pharmacological Research.

[51] Lu X, Su N, Yang J, Huang W, Li C, Zhao L (2009). Fibroblast growth factor receptor 1 regulates the differentiation and activation of osteoclasts through Erk1/2 pathway. Biochemical and biophysical research communications.

[52] Pagès G, Guérin S, Grall D, Bonino F, Smith A, Anjuere F (1999). Defective thymocyte maturation in p44 MAP kinase (Erk 1) knockout mice. Science (New York, NY).

[53] Yao Y, Li W, Wu J, Germann UA, Su MS, Kuida K (2003). Extracellular signal-regulated kinase 2 is necessary for mesoderm differentiation. Proceedings of the National Academy of Sciences of the United States of America.

[54] Vantaggiato C, Formentini I, Bondanza A, Bonini C, Naldini L, Brambilla R (2006). ERK1 and ERK2 mitogen-activated protein kinases affect Ras-dependent cell signaling differentially. J Biol.

[55] Milde-Langosch K (2005). The Fos family of transcription factors and their role in tumourigenesis. European journal of cancer (Oxford, England: 1990).

[56] Hu E, Mueller E, Oliviero S, Papaioannou VE, Johnson R, Spiegelman BM (1994). Targeted disruption of the c-fos gene demonstrates c-fos-dependent and -independent pathways for gene expression stimulated by growth factors or oncogenes. The EMBO journal.

[57] Chiu R, Boyle WJ, Meek J, Smeal T, Hunter T, Karin M (1988). The c-Fos protein interacts with c-Jun/AP-1 to stimulate transcription of AP-1 responsive genes. Cell.

[58] Shaulian E (2010). AP-1-The Jun proteins: Oncogenes or tumor suppressors in disguise?. Cell Signal.

[59] Fan DM, Feng XS, Qi PW, Chen YW (2014). Forkhead factor FOXQ1 promotes TGF-beta1 expression and induces epithelial-mesenchymal transition. Molecular and cellular biochemistry.

[60] Christensen J, Bentz S, Sengstag T, Shastri VP, Anderle P (2013). FOXQ1, a novel target of the Wnt pathway and a new marker for activation of Wnt signaling in solid tumors. PloS one.

[61] Zhang J, Li W, Dai S, Tai X, Jia J, Guo X (2015). FOXQ1 is overexpressed in laryngeal carcinoma and affects cell growth, cell cycle progression and cell invasion. Oncol Lett.

