# Supplementary material for: The FGFR1 Signaling Pathway Upregulates the Oncogenic Transcription Factor FOXQ1 to Promote Breast Cancer Cell Growth
Source: Int J Biol Sci. 2023 Jan 1;19(3):744–59. doi: 10.7150/ijbs.74574 (PMC9909991; doi:10.7150/ijbs.74574)
Supplement: Supplementary file 1 — Supplementary figures and tables. [file ijbsv19p0744s1.pdf]

Supplementary Materials

Table S1. siRNA sequences

siRNA	Sequence	
	Sense (5'-3')	antisense (5'-3')
siFOXQ1-1	CCAUCAAACGUGCCUUAAATT	UUUAAGGCACGUUUGAUGGTT
siFOXQ1-2	CGCGGACUUUGCACUUUGATT	UCAAAGUGCAAAGUCCGCGTT
siNC	UUCUCCGAACGUGUCACGUTT	ACGUGACACGUUCGGAGAATT

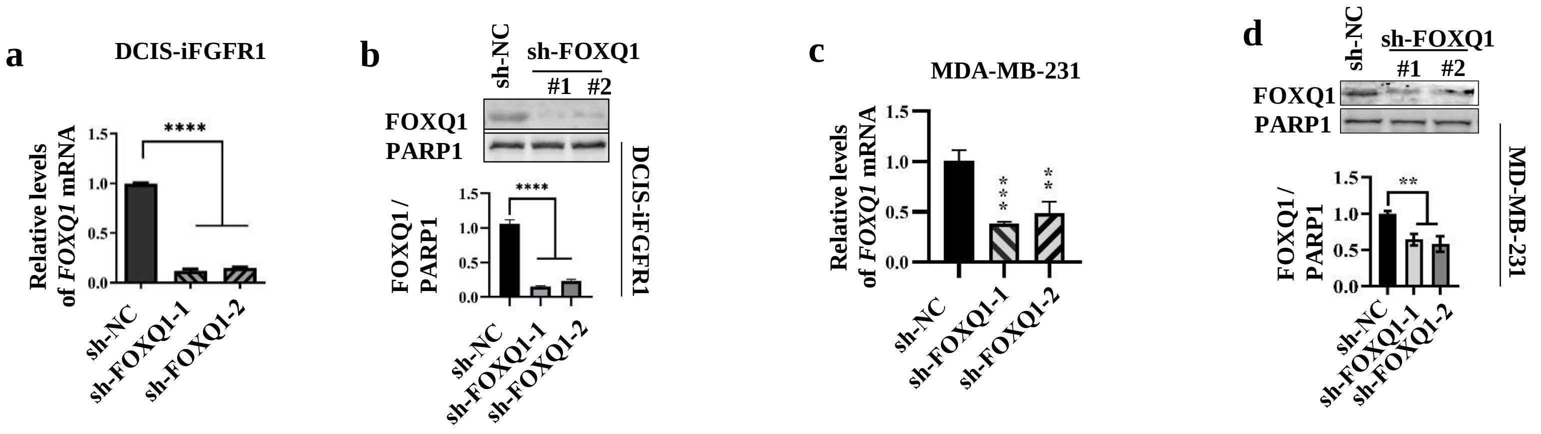

Fig. S1. Generation of *FOXQ1* knockdown DCIS-iFGFR1 and MDA-MB-231 cells. a and **b**. RT-qPCR and Western blot (WB) assays of FOXQ1 mRNA and protein in sh-NC control, sh-FOXQ1-1, and sh-FOXQ1-2 expressing DCIS-iFGFR1 cell lines. **c** and **d**. RT-qPCR and WB assays of FOXQ1 mRNA and protein in sh-NC control, sh-FOXQ1-1, and sh-FOXQ1-2 expressing MDA-MB-231 cell lines. In all panels, quantitative data were obtained from 3 independent assays. The relative expression levels of FOXQ1 mRNA and protein were normalized to β-actin mRNA and PARP1 protein, respectively. **, ***, and ****, $p < 0.01$, 0.001, and 0.0001, respectively by One-Way ANOVA test.

Table S2. Transcription factors predicted to bind to the FOXQ1 promoter by the PROMO database

Name	Matrix	Width	Name	Matrix	Width	Name	Matrix	Width
GR-α	[T00337]	5	EBF	[T05427]	11	GATA-1	[T00306]	6
C/EBPβ	[T00581]	4	AP-1	[T00029]	9	VDR	[T00885]	9
Pax-5	[T00070]	7	c-Jun	[T00133]	7	PXR-1	[T05671]	8
p53	[T00671]	7	c-Fos	[T00123]	10	GR	[T05076]	7
GR-β	[T01920]	5	T3R-β1	[T00851]	9	TFIID	[T00820]	7
NF-kappaB1	[T00593]	11	IRF-2	[T01491]	6	Sp1	[T00759]	10
IRF-1	[T00423]	9	PPAR-α	[T05221]	11	C/EBPα	[T00105]	7
ENKTF-1	[T00255]	8	ETF	[T00270]	11	HNF-3α	[T02512]	8
TFII-I	[T00824]	6	RAR-β	[T00721]	10	NFI/CTF	[T00094]	8
STAT4	[T01577]	6	E2F-1	[T01542]	8	GCF	[T00320]	9
c-Ets-1	[T00112]	7	WT1	[T00899]	9	MAZ	[T00490]	13
NF-1	[T00539]	8	Egr-1	[T00241]	16	HOXD9	[T01424]	10
AP-2αA	[T00035]	6	Sp3	[T02338]	16	HOXD10	[T01425]	10
YY1	[T00915]	4	Elk-1	[T00250]	9	SRY	[T00997]	9
RXR-α	[T01345]	7	uSF2	[T00878]	10	TCF-4E	[T02878]	7
FOXP3	[T04280]	6	LEF-1	[T02905]	8	c-Ets-2	[T00113]	9
PR B	[T00696]	7	ER-α	[T00261]	5	XBP-1	[T00902]	6
PR A	[T01661]	7	TCF-4	[T02918]	10	AR	[T00040]	9
						NF-Y	[T00150]	8

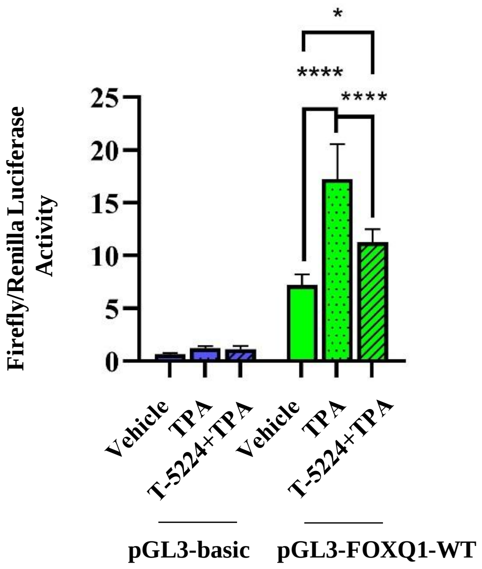

Fig. S2. c-FOS strongly increases the the transcriptional activity of the *FOXQ1* promoter. HEK293 cells were transfected with pGL3-basic vector or FOXQ1-WT reporter with a firefly luciferase sequence. Another Renilla luciferase-expressing vector was co-transfected as a normalizer. Transfected cells were treated with vehicle, TPA, or TPA and T-5224 as indicated. The relative activities of the pGL3-basic control and the FOXQ1-WT firefly luciferase reporters were presented after normalized to the Renilla luciferase activity. * and ****, $p < 0.05$ and 0.0001, respectively by the One-Way ANOVA test.
